# *Plasmodium vivax* ligand-receptor interaction: *Pv*AMA-1 domain I contains the minimal regions for specific interaction with CD71+ reticulocytes

**DOI:** 10.1038/s41598-017-10025-6

**Published:** 2017-08-30

**Authors:** Gabriela Arévalo-Pinzón, Maritza Bermúdez, Diana Hernández, Hernando Curtidor, Manuel Alfonso Patarroyo

**Affiliations:** 1 Fundación Instituto de Inmunología de Colombia (FIDIC), Carrera 50 # 26-20, Bogotá, Colombia; 2 PhD Program in Biomedical and Biological Sciences, Universidad del Rosario, Carrera 24 #, 63C-69 Bogotá, Colombia; 3 MSc Program in Biological Sciences, Pontificia Universidad Javeriana, Carrera 7 # 40-62, Bogotá, Colombia; 4 School of Medicine and Health Sciences, Universidad del Rosario, Carrera 24 #, 63C-69 Bogotá, Colombia

## Abstract

The malarial parasite’s invasion is complex, active and coordinated, involving many low and high affinity interactions with receptors on target cell membrane. Proteomics analysis has described around 40 proteins in *P. vivax* which could be involved in reticulocyte invasion; few have been studied with the aim of elucidating how many of them establish specific interactions with their respective host cells. Given the importance of knowing which of the parasite’s protein regions are functionally important for invasion, minimum regions mediating specific interaction between *Plasmodium vivax* apical membrane antigen 1 (*Pv*AMA-1) and its host cell were here elucidated. The region covering *Pv*AMA-1 domains I and II (*Pv*AMA-DI-II) specifically bound to the CD71^+^ red blood cell subpopulation. A 20 residue-long region (^81^EVENAKYRIPAGRCPVFGKG^100^) located in domain I was capable of inhibiting *Pv*AMA-DI-II recombinant protein binding to young reticulocytes (CD71^+^CD45^−^) and rosette formation. This conserved peptide specifically interacted with high affinity with reticulocytes (CD71^+^) through a neuraminidase- and chymotrypsin-treatment sensitive receptor. Such results showed that, despite AMA-1 having universal functions during late *Plasmodium* invasion stages, *Pv*AMA-1 had reticulocyte-preferring binding regions, suggesting that *P. vivax* target cell selection is not just restricted to initial interactions but maintained throughout the erythrocyte invasion cycle, having important implications for designing a specific anti-*P. vivax* vaccine.

## Introduction

*Plasmodium vivax* is one of five species causing human malaria; it is responsible for more than half the cases reported outside Africa, accounting for ~100 million cases and around 2.5 billion people at risk of infection^1, 2^. In spite of this, worldwide malaria control strategies and advances regarding vaccine design have mainly been focused on *P. falciparum* ^3^. However, clinical, epidemiological and biological differences between these two species regarding infection severity, blood phase dynamics, geographical distribution, type of target cell and disease transmission dynamics^2–4^ suggest that *P. falciparum*-related control mechanisms might not be effective against *P. vivax* and provide an initiative for in-depth research concerning *P. vivax* biology directed towards developing drugs and vaccines against this species.

One of the main thrusts of research is concerned with elucidating the repertoire of receptor-ligand complexes used by *P. vivax* to enter host cells. Four steps define multistep erythrocyte invasion; there is initial contact with the host cell followed by merozoite (Mrz) reorientation of the apical pole to ensure direct contact with the membrane, leading to high affinity interactions being established for forming a strong bond/tight junction (TJ) acting as anchor so that myosin actin motor can enable parasite sliding within a nascent parasitophorous vacuole in which it resides and multiplies^5^. Merozoite surface protein-1 (MSP-1) interaction with heparin^6^ has been described for *P. falciparum*, followed by its interaction with band 3^7^ and glycophorin A on erythrocyte surface^8^ which is continued by interaction between erythrocyte binding antigens (EBA-175, EBA-140, EBA-181 and EBL-1) and/or reticulocyte-binding-like homologues (*Pf*Rh2a, *Pf*Rh2b and *Pf*Rh4) with receptors such as glycophorin A^9^, glycophorin C^10^, protein 4.1^11^, glycophorin B^12^ and complement receptor-1 (CR1)^13^. EBA and Rh proteins define one of the main *P. falciparum* evasion mechanisms depending on receptor availability and/or changes in the expression of such antigens, forcing the parasite to alternate between invasion pathways^14, 15^. Afterwards, Rh5 interaction with Basigin triggers rhoptry content release associated with calcium flux on parasite/host interface^5^, followed by an interaction between apical membrane antigen-1 (AMA-1) and rhoptry neck protein-2 (RON2) leading to TJ formation, facilitating parasite penetration^16^.

Such host-parasite interactions are poorly understood in *P. vivax*. The main interactions evaluated regarding this species are orientated towards proteins involved in Duffy-positive reticulocytes’ initial contact and selection as target cells. Research published in 1994 showed that Duffy binding protein (DBP) region II was involved in interaction with the Duffy antigen receptor for chemokines (DARC)^17^ while Galinski *et al*., described two members from the reticulocyte binding protein (RBP) family (RBP-1 and RBP-2) having reticulocyte specificity and binding to yet-to-be-defined receptors^18^. Moreover, *Pv*MSP-1 also contains specific regions involved in interaction with reticulocytes^19^. *P. vivax* genome sequencing has revealed new RBP family members believed to provide recognition and specificity in reticulocyte binding^4^ and which could be involved in an alternative pathway in an attempt to justify the presence of *P. vivax* in Duffy-negative patients^20^. However, flow cytometry-based erythrocyte-binding assays have shown that *Pv*RBP2a recognizes a receptor on both reticulocytes and mature erythrocytes^21^ while RBP1a (known earlier as RBP1) and RBP1b recombinant proteins only bind to reticulocytes^22^; on the contrary, another study showed that RBP1a and RBP1b displayed preferential normocyte binding^21^. A novel protein named erythrocyte binding protein-2 (EBP-2), representing a new member of the DBP family, preferentially binds to young (CD71^high^) Duffy-positive reticulocytes and has minimal Duffy-negative reticulocyte binding capability, suggesting that EBP-2 mediates a Duffy-independent infection pathway^23^. Recent research has highlighted a molecule expressed in rhoptries named *Pv*RON5 which, despite binding to all RBC, displayed a marked preference for human reticulocytes^24^.

AMA-1, one of the leading blood stage vaccine candidates involved in later contact events during *P. falciparum* Mrz and sporozoite (Spz) invasion, was selected to increase knowledge about specific interactions between *P. vivax* and reticulocytes^25, 26^. AMA-1 is a micronemal type I transmembrane protein which is translocated to parasite surface via the rhoptry neck just prior to or during host cell invasion and is conserved among apicomplexan parasites^27, 28^. This protein forms the ectodomain within which 16 conserved cysteines contribute to eight disulphide bonds folding the protein into three major domains (DI; DII and DIII)^29^. *Plasmodium spp*. and *T. gondii* AMA-1 crystal structures have shown that core domains I and II are based on the Plasminogen Apple Nematode (PAN) folding motif, defining a superfamily of protein folding implicated in receptor binding^30^ and hydrophobic pocket formation^31, 32^.

The 83 kDa precursor protein (*Pf*AMA-1_83_) becomes converted to *Pf*AMA-1_66_^33^ during Mrz release and erythrocyte invasion; this protein undergoes two C-terminal cleavages during invasion, giving rise to 48-kDa and 44-kDa soluble forms^34^. No viable parasites were obtained when the gene encoding *Pf*AMA-1 was disrupted in *P. falciparum* asexual blood stages and/or other *Plasmodium* species, indicating that the encoded protein has an essential function during this part of the parasite cycle^27, 35^. Several studies have implicated *Plasmodium* AMA-1 in erythrocyte binding^36–38^ as well as *P. knowlesi* Mrz reorientation on RBC surface^39^ and more recently in mediating TJ formation together with rhoptry-derived proteins (RON proteins)^16, 40^. *Pf*AMA-1 conditional expression during the intra-erythrocyte cycle has directly or indirectly involved this protein in resealing erythrocytes at the end of invasion^41^. *T. gondii Tg*AMA-1-depleted tachyzoites have significant defects regarding rhoptry secretion, compromising their ability to invade host cells^42^.

Even though it is still not completely clear how this protein mediates and integrates all these tasks during invasion, monoclonal antibodies or synthetic peptides binding close to or in the *Pf*AMA-1 hydrophobic pocket inhibit erythrocyte invasion, some of them blocking interaction between *Pf*AMA-1 and *Pf*RON2^43–46^. Immunization with AMA-1 induced invasion-inhibiting antibodies, conferring protection in animals^47–49^; however, this protection has shown to be strain specific. In fact, immunizing mice with *P. chabaudi* DS strain-derived AMA-1 has conferred complete protection against homologous challenge but poor protection has been observed regarding challenge with *P. chabaudi* 556KA strain^50^. Likewise, naturally-acquired antibodies against *Pf*AMA-1 has strongly inhibited 3D7 strain Mrz invasion but has had a poor effect on other *P. falciparum* strains^51^. Naturally-acquired immunity to *Pv*AMA-1 is associated with the appearance of cytophilic antibodies (IgG1 and IgG3) related to protection^52^. Different AMA-1 formulations found during vaccine clinical development have been based on one or two *P. falciparum* strains inducing strain-specific protective responses^53, 54^ or, in some cases, the vaccine has not been efficient even against homologous parasites^55^. Although AMA-1 is the major target in naturally-acquired invasion inhibitory antibodies, this protein has a high degree of allelic diversity^56, 57^; several studies analyzing AMA-1 sequences from different geographical regions have shown that the *ama-1* gene is under balancing selection, thereby posing a challenge when designing a vaccine based on this antigen^58–61^. Even though different authors have suggested including multiple alleles in a vaccine to induce antibodies having wide-scale reactivity and thus covering the parasite population’s global genetic diversity^62, 63^, it is also important to ascertain which AMA-1 regions are involved in this protein’s vital functions to guide any immune response towards these regions thereby leading to developing control methods covering *Plasmodium*’s broad allele spectrum.

Within the framework of developing a specific vaccine against *P. vivax* there is enormous interest in identifying and characterizing the functional binding regions which this parasite uses to invade its target cells. Taking into account the important experimental antecedents mentioned above, a series of experiments was thus carried out; this led to identifying a conserved region of *Pv*AMA-1 located in domain I (DI) specifically interacting with reticulocytes which could be used as template for inclusion in a synthetic multistage, multi-antigen vaccine against *P. vivax*.

## Results

### *Pv*AMA-DI-II bound to total RBC

**Figure 1:**
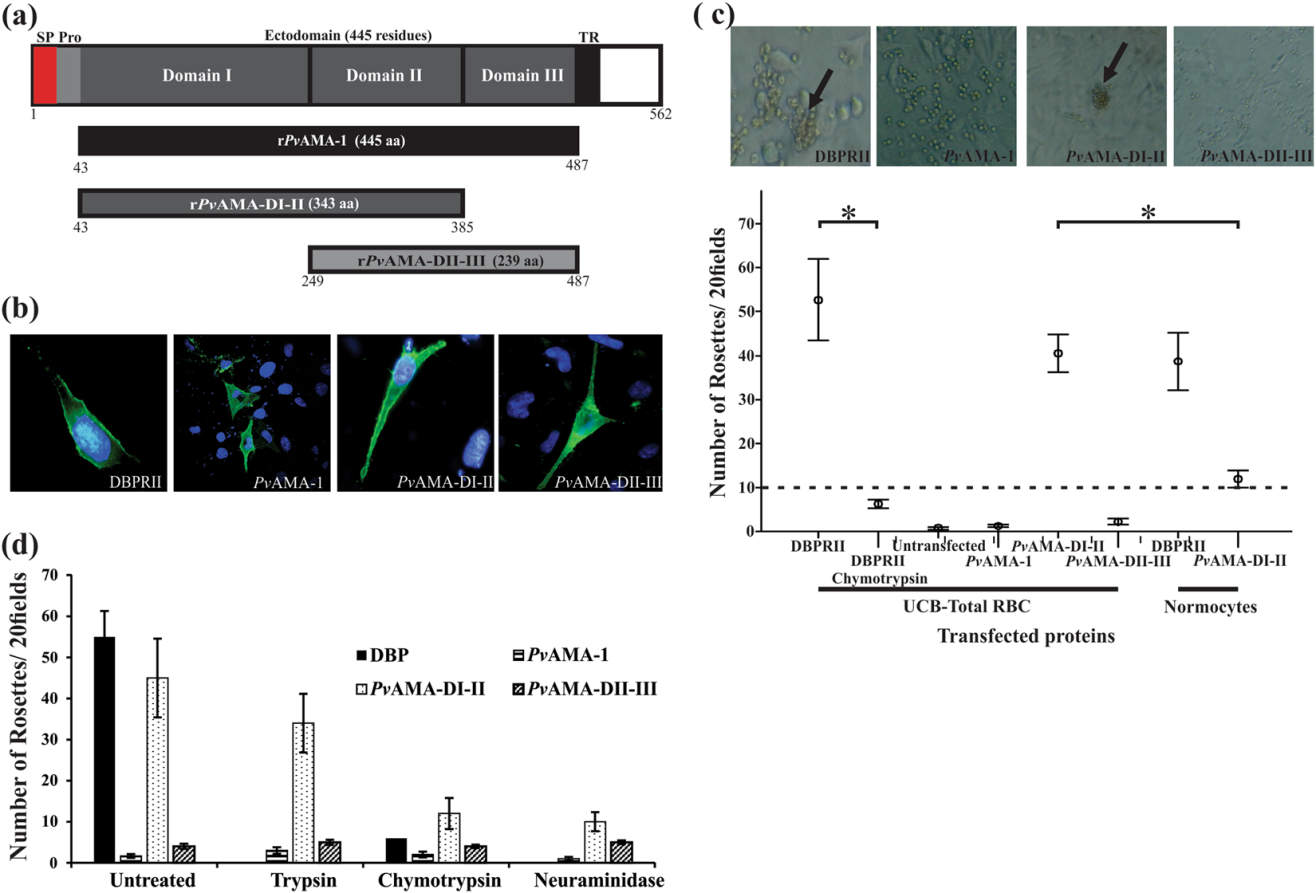
COS7 cells expressing *Pv*AMA*-*DI-DII bound to UCB RBC. (**a**) Schematic representation of *Pv*AMA-1 primary structure. Signal peptide (SP) is indicated in red, pro-sequence (Pro) in light grey, ectodomain (consisting of domain I (DI), domain II (DII) and domain III (DIII)) in dark grey, transmembrane region (TR) in black and cytoplasmic tail in white. The three fragments were expressed both on COS-7 cell membrane and as soluble recombinant proteins in *E. coli*. Black shows the complete *Pv*AMA-1 ectodomain (r*Pv*AMA-1), dark grey represents domains I-II (r*Pv*AMA*-*DI-II) and light grey domains II–III (r*Pv*AMA-DII-III). The first and last residues in each region are indicated. (**b**) Immunofluorescence assay. Each of the three *Pv*AMA-1 fragments was cloned in the pRE4 vector and expressed on COS-7 cell membrane. The presence of HSV gD protein signal peptide and transmembrane domain in pRE4 vector enabled the expression of each fragment on cells surface, as shown by green fluorescence. Cell nuclei were stained with DAPI. (**c**) Rosette formation assays. The binding assay was performed with UCB RBC or reticulocyte-depleted normocytes. Only *Pv*AMA-DI-DII and DBPRII transfected cells could form rosettes (black arrows).The amount of rosettes for each protein expressed on COS-7 cells is shown. DBPRII was used as positive control while non-transfected cells and DBPRII binding to chymotrypsin-treated UCB were used as negative controls. (**d**) Trypsin-, chymotrypsin- and neuraminidase-treated UCB RBC binding assays. It can be seen that the amount of rosettes became reduced with *Pv*DI-DII protein when RBC were treated with chymotrypsin and neuraminidase. Only intact erythrocyte and chymotrypsin-treated DBPRII binding was evaluated. At least three independent experiments (with replicates) were performed for all experiments. The error bars indicate the standard deviation.

The full *Pv*AMA-1 ectodomain, *Pv*AMA-DI-II and *Pv*AMA-DII-III (Fig. 1a) were expressed on the COS-7 cell surface (Fig. 1b) to evaluate whether *Pv*AMA-1 bound to human RBC. The herpes simplex virus (HSV) gD gene’s signal peptide and transmembrane domain, included in pRE4 mammalian expression vector^64^, allowed different *Pv*AMA-1 constructs to become translocated to the surface of transfected COS-7 cells. Immunofluorescence assays using non-permeabilized transfected COS-7 using DL6^17^ or F8.12.19^65^ antibodies confirmed these proteins’ correct orientation on COS-7 cell surface (Fig. 1b); no staining with these antibodies was observed in non-transfected cells. Transfection efficiency was seen to be 4%-5% for all constructs, including transfection of the pHVDR22 plasmid which expresses DBPRII used as a positive control^17^ (Fig. 1b), agreeing with transfection efficiency obtained in other studies with *Plasmodium* antigens^66, 67^. The transfected cells incubated with umbilical cord blood (UCB) (about 5–7% of reticulocytes) showed that only *Pv*AMA-1 domains I and II (*Pv*AMA-DI-II) bound to RBC, although the amount of rosettes was lower than for the DBPRII construct (p > 0.05) (Fig. 1c). Negative controls with either non-transfected COS-7 with human RBC or COS-7 expressing DBPRII with chymotrypsin-treated RBC (which removes the Duffy antigen receptor)^68^ gave no rosettes or significantly decreased (p < 0.05) the amount of rosettes compared to untreated RBC (Fig. 1c). No significant amount of rosettes was found for the full *Pv*AMA-1 ectodomain or *Pv*AMA-DII-III, suggesting that the domain I and II region is responsible for *Pv*AMA-1 binding. *Pv*AMA-DI-II binding was affected regarding neuraminidase- or chymotrypsin-treated RBC, being reduced by 71% or 73%, respectively, while limited impact on binding was observed concerning trypsin-treated RBC (Fig. 1d). Enzyme treatment of RBC led to no significant change in the amount of rosettes for either of the other two *Pv*AMA-1 fragments, different from what has been reported for *Pf*AMA-1 domain III which only binds to its receptor (known as Kx) on trypsin-treated erythrocytes^38^.

Taking into account that *P. vivax* preferentially infects reticulocytes, an evaluation was made of whether *Pv*AMA-DI-II binding concerns interaction with all RBC populations or only a minor subpopulation (reticulocytes). As purified intact reticulocytes (>99%) could not be obtained, peripheral blood (containing only 1% reticulocytes) depleted in white blood cells and reticulocytes was used to obtain only normocytes and determine binding properties regarding the latter cells. The amount of rosettes decreased with normocytes regarding UCB (p = 0.01) for COS-7 expressing *Pv*AMA-DI-II (Fig. 1d), suggesting that these domains bound to a specific non-normocyte RBC sub-population while only a slight reduction in the el amount of rosettes was observed for DBP region II thereby agreeing with that published previously for this region^23^.

### CD71^+^ cells were the main *Pv*AMA-DI-II binding targets

**Figure 2:**
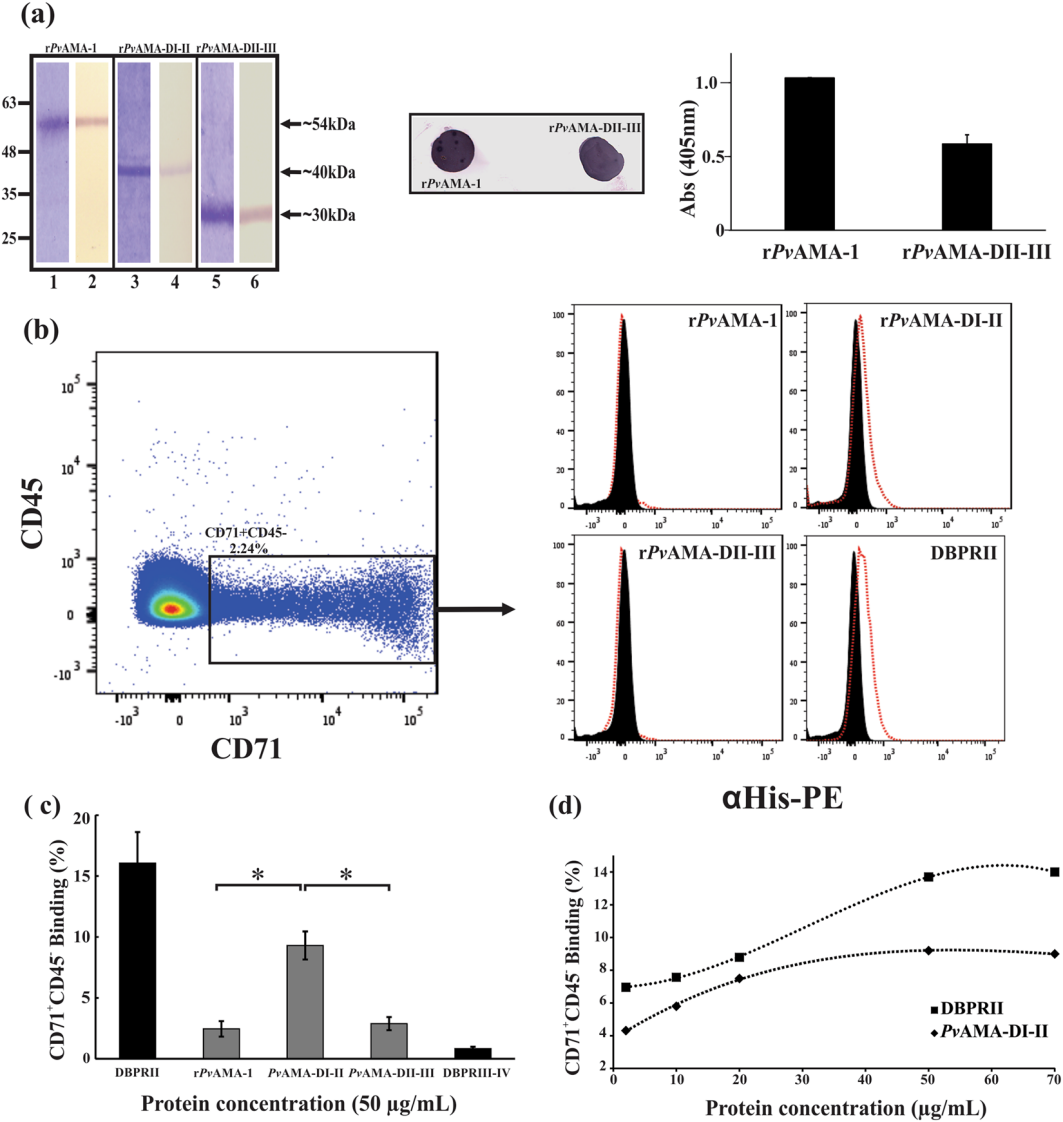
Soluble recombinant protein *Pv*AMA*-*DI-II only interacted with CD71^+^ reticulocytes. (**a**) Three soluble *Pv*AMA-1 recombinant fragments were obtained and purified by affinity chromatography. Lanes 1, 3 and 5 show recombinant proteins resolved by SDS-PAGE followed by Coomassie blue staining. A single expected molecular weight band was observed for each protein fragment. Lanes 2, 4 and 6 show Western blot recognition of each recombinant protein by anti-histidine tag monoclonal antibody. (**b**) Dot plot showing the CD71^+^CD45^**−**^ study population (black box). This population was used for determining each recombinant protein’s binding. Histograms showing each recombinant protein’s binding to CD71^+^CD45^**−**^ cells (reticulocytes). Each protein’s cell binding was detected with PE conjugated anti-His Tag antibodies. Displacement (red lines) regarding control was observed in r*Pv*AMA*-*DI-II and DBPRII histograms. (**c**) CD71^+^CD45^**−**^ cell binding percentages for each protein evaluated on the X axis. At least three independent experiments (with replicates) were performed for all experiments. The error bars indicate the standard deviation. Soluble recombinant protein DBPRII was used as positive binding control and DBPRIII-IV as negative binding control. (**d**) Saturation assay showing r*Pv*AMA*-*DI-II and DBPRII total binding at different protein concentrations.

Previous studies have shown that immature reticulocytes (CD71^**+**^, I-II-III stages) are the major *P. vivax* target cells, whereas older CD71^**−**^ (IV stage) reticulocytes were rarely invaded^69^. A cytometry-based erythrocyte-binding assay was thus used to evaluate whether *Pv*AMA-DI-II binding was dependent on a specific RBC subpopulation (i.e. reticulocytes (CD71^+^). Three soluble recombinant proteins (r*Pv*AMA-1, r*Pv*AMA-DI-II and r*Pv*AMA-DII-III) (Fig. 1a) were obtained in *E. coli* and purified by affinity chromatography (Fig. 2a). These proteins displayed a molecular mass of ~54 kDa (r*Pv*AMA-1), ~40 kDa (r*Pv*AMA-DI-II) and ~27 kDa (r*Pv*AMA-DII-III) in SDS-PAGE, and were recognized by WB with a monoclonal anti-histidine antibody (Fig. 2a). r*Pv*AMA-1 and r*Pv*AMA-DII-III were recognized by mAb F8.12.19 in dot blot and ELISA assays, confirming correct recombinant protein folding (Fig. 2a). This mAb recognized a conformational epitope on full-*Pv*AMA-1, specifically in domain III^65^.

Each recombinant protein was incubated with UCB and reticulocytes were stained with transferrin receptor (CD71) antibodies; this receptor is known to be expressed during the earliest erythroid precursor stages and becomes progressively depleted during reticulocyte maturation into normocytes^70^. It has also been found in activated lymphocytes^71^; an antibody against the CD45 marker (found in all leukocytes) having greater expression in lymphocytes was thus included to rule out this population from the binding study (Supplementary Fig. S1). The study population thus consisted of CD71^+^CD45^−^ cells (mean 2.07%: 3.58% maximum and 1.54% minimum) (Fig. 2b) corresponding to the population of preference in *P. vivax* invasion. Each recombinant protein’s binding to CD71^+^CD45^−^ cells was detected with a phycoerythrin-conjugated anti-histidine monoclonal antibody. The histograms show that soluble recombinant DBPRII bound to CD71^+^CD45^−^cells *(*16.1% ± 2.5) as did r*Pv*AMA-DI-II (9.3% ± 1.2) whereas only 2.5% ± 0.6 binding was found for r*Pv*AMA-1 and 2.9 ± 0.5 for r*Pv*AMA-DII-III (p < 0.05) (Fig. 2b and c). Only 0.8% binding of DBPRIII-IV was found; earlier studies have shown that this fragment did not bind to either normocytes or reticulocytes, so it was considered as a negative control^17, 72^. r*Pv*AMA-DI-II binding activity was shown to increase in a concentration-dependent manner and was saturated at 50 µg/mL, similar to DBPRII (Fig. 2d). Only 0.03% to 0.3% binding was found when each *Pv*AMA-1 recombinants’ binding to the CD71^−^CD45^−^ population (normocytes and stage IV reticulocytes) was evaluated (Supplementary Fig. S2). 3.4% ± 0.5 binding was found for *Pv*DBP RII thereby agreeing with previous work showing that DBPRII-DARC interaction is more abundant with reticulocytes than total RBC^23^.

### Conserved peptide 21270 had binding properties which were CD71^+^ reticulocyte-specific for the *Pv*AMA-DI-II región

**Figure 3:**
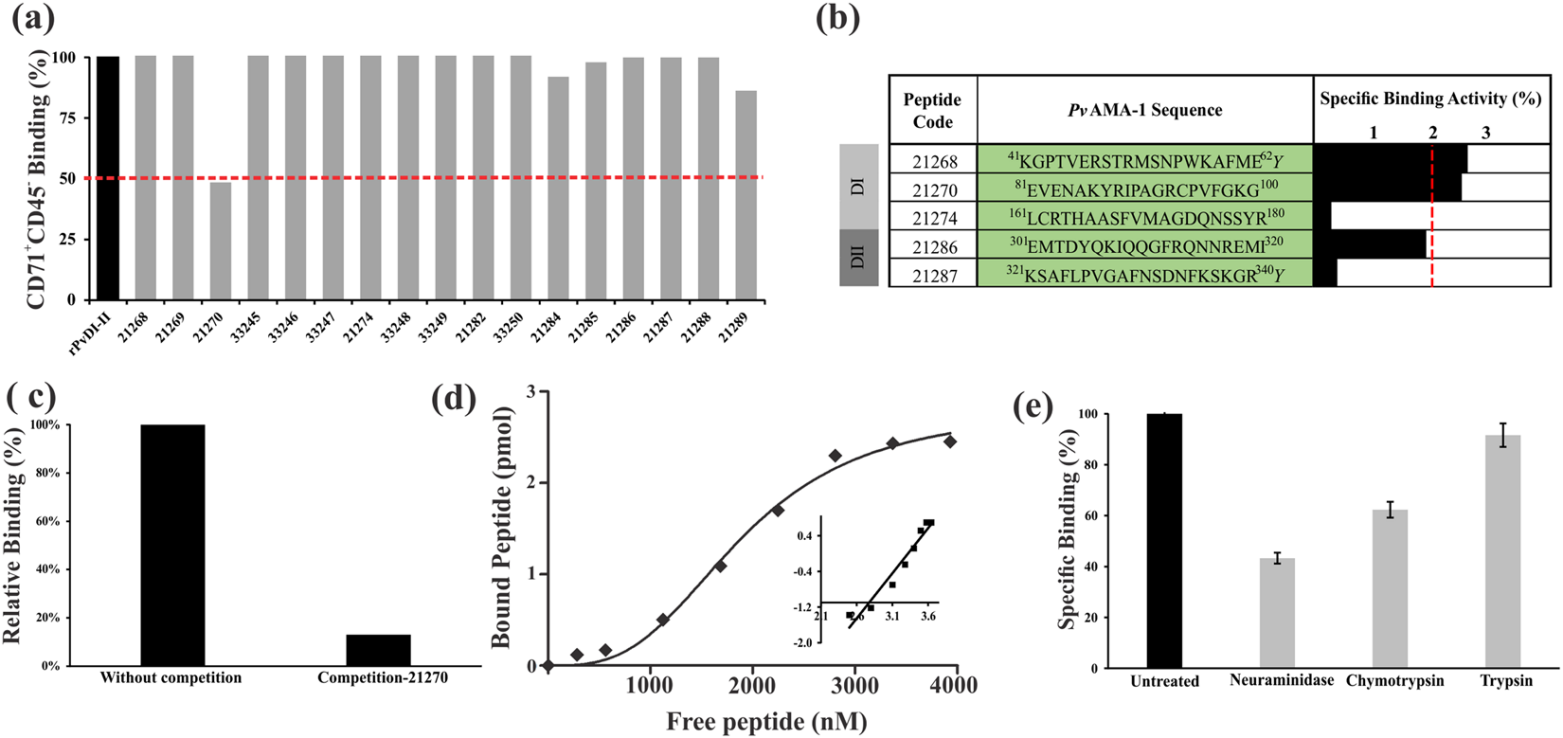
*Pv*AMA-1 domain I contained peptides specifically interacting with reticulocytes. (**a**) Flow cytometry inhibition experiments. 20 residue-long synthetic peptides covering the complete *Pv*AMA-1 domain I and II sequence were used. Peptide numbers were assigned according to FIDIC’s serial numbering system. Each peptide was pre-incubated with UCB RBC and then incubated with r*Pv*AMA-DI-II. Recombinant protein binding percentages were detected with PE conjugated anti-Histidine tag antibodies. (**b**) Binding profile of conserved peptides within *Pv*AMA*-*1 domains I-II to UCB RBC. Black bars represent percentage binding activity defined as the amount of peptide (picomoles) binding specifically per added peptide (picomoles). Peptides having ≥2 binding percentage (red line) were considered HABPs, according to previously described criteria^74^. Each assay was carried out in triplicate. A tyrosine residue was added at the C-terminus of peptides 21268 and 21287 to enable radiolabeling. (**c**) Inhibition experiments involving rosetting assay. Peptide 21270 was pre-incubated with UCB RBC; RBC were then incubated with COS-7 cells expressing the *Pv*AMA*-*DI-II region. The values show *Pv*AMA*-*DI-II average relative UCB RBC binding percentage in the absence (100%) or presence of peptide 21270. (**d**) HABP 21270 saturation curves. The curve represents specific binding. The x-axis in the Hill plot (inset) shows log F = free peptide and the y-axis log (B/Bmax−B), where B is the amount of bound peptide and Bmax the maximum amount of bound peptide. (**e**) The effect of enzyme treatment on HABP 21270 binding to RBC. Each bar represents peptide 21270 specific binding activity percentages upon incubation with neuraminidase-, chymotrypsin- and trypsin-treated RBC. Untreated RBC were used as control (black). Standard deviations were below 5%.

Seventeen peptides covering the whole *Pv*AMA-1 domains I and II (residues 41 to 380) were chemically synthesized and included in a CD71^+^ cell binding inhibition experiment where each peptide competed with r*Pv*AMA-DI-II to delineate the specific region within *Pv*AMA-DI-II involved in CD71^+^ reticulocyte interaction. Figure 3a shows that adding peptide 21270 (located in DI) produced a 52% reduction regarding r*Pv*AMA-DI-II binding to CD71^+^CD45^−^cells and 14% concerning 21289 (located in DII).

We have previously reported a sensitive and specific binding test for detecting receptor-ligand interactions between peptides and receptors on RBC surface^73^. Each synthetic peptide derived from malarial Spz and Mrz antigens was radiolabeled and incubated with human hepatocytes or RBC in the presence or absence of the same unlabeled peptide. The specific binding plot was obtained from the difference between total binding (binding in the absence of non-radiolabeled peptide) and unspecific binding (binding in the presence of non-radiolabeled peptide) plots^74^. Peptides having ≥0.02 slope specific binding plot were considered high activity binding peptides (HABPs) and used as templates for designing an anti-malarial vaccine^75^. Some HABPs were conserved (no amino acid sequence variation) and others had genetic variability and elicited a strain-specific immune response allowing malarial parasites to escape vaccine-induced immunity, meaning that they were discarded from further immunological studies^74^.

Taking into account that AMA-1 is significantly polymorphic in all *Plasmodium* species^60, 76^, the specific binding activity of *Pv*AMA-1 peptides which did not have amino acid changes was measured (Supplementary Fig. S2) to describe this protein’s conserved functional regions involved in interaction with specific target cells. Five conserved peptides were selected, three of them in DI and two in DII, peptides 21268 and 21270 being found to be HABPs having ≥2% specific binding activity (Fig. 3b). Only 0.7% to 1.2% specific binding (not surpassing the ≥2% threshold) was found when these peptides were assayed for normocyte binding showing that the specific binding activity measured for these two peptides was directed towards their interaction with reticulocytes, as already shown by cytometry (Fig. 2).

Due to HABP 21270 being able to inhibit recombinant protein interaction by more than 50% (1:20 molar ratio) (Fig. 3a), it was evaluated whether it could inhibit rosette formation. Adding UCB RBCs pre-incubated with peptide 21270 to COS-7 cells expressing *Pv*AMA-DI-II inhibited the amount of rosettes by 87% compared to control (Fig. 3c). Measuring HABP 21270 binding to UCB RBC at different radiolabeled peptide concentrations in the presence or absence of unlabeled peptide yielded a 1.9 ± 0.34 µM *K*_d_, Hill coefficient = 3.0 ± 0.47 indicating positive cooperativity (Fig. 3d).

HABP 21270-CD71^+^ reticulocyte interaction was sensitive to treatment with neuraminidase and chymotrypsin (Fig. 3e), similar to the pattern observed for the whole of domains I-II, suggesting that this peptide located in domain I had *Pv*AMA-1-specific binding properties.

### *Pv*AMA-1 HABPs were located near the hydrophobic binding Groove

**Figure 4:**
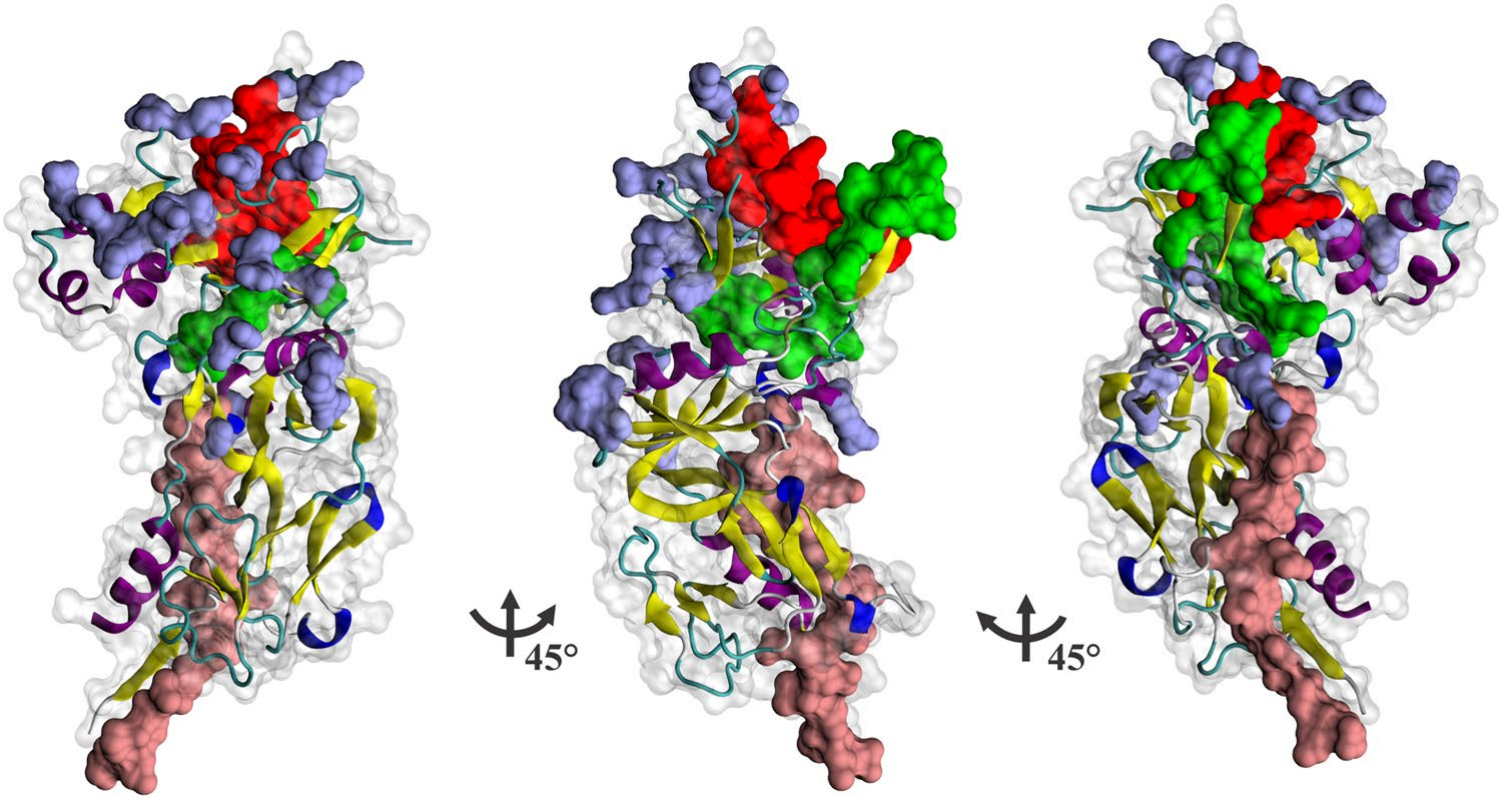
HABP 21270 formed part of the *Pv*AMA-1 hydrophobic trough. Surface view of the *Pv*AMA-1 ectodomain, showing HABP 21268 (pink) and HABP 21270 (green) localization. Red shows part of the hydrophobic trough formed between both PAN domains and grey shows the cluster of polymorphic residues in *Pv*AMA-1 contained in domain I and II^61^. The molecule’s secondary structure is shown, yellow highlighting the *β*-sheets, violet the *a*-helices, blue the turns and white shows random structure. This representation was built in the VMD (Visual Molecular Dynamics) software, taking PDB 1W81 as source^31^.

The *Pv*AMA-1 structure was solved by Pizarro *et al*.^31^, who described three domains comprising the antigen’s ectodomain. When HABPs 21268 and 21270 were located on *Pv*AMA-1 structure it was found that both peptides were located on the same face (Fig. 4), opposite to the face where most polymorphic sites which contribute towards *Pv*AMA-1’s overall diversity^61^ were found.

## Discussion

Determining *P. vivax* antigens’ functional regions could be one of the best strategies for blocking this parasite’s biological functions during host cell entry. Comparative studies involving *P. falciparum* have led to a significant amount of new antigens being identified in *P. vivax* ^77^; in spite of this, few studies have emphasized the identification of *P. vivax* proteins’ functional regions during invasion, mainly due to this species preferentially invading reticulocytes (low concentration in peripheral blood) together with the inherent characteristics in each *P. vivax* strain impeding the development of a continuous culture *in vitro* ^78^. Even though short-term cultures have been developed, it has been observed that parasite density becomes reduced as intra-erythrocytic cycles advance, even when adding fresh reticulocytes^79^. The foregoing, plus variability concerning parasitemia percentages depending on the isolate^79, 80^, could lead to a poor interpretation of the role played by a particular antigen during *P. vivax* Mrz invasion.

Evaluating some *P. vivax* proteins’ binding to human reticulocytes represents an approach to begin describing the possible antigens involved in host cell invasion. Some regions having specific binding to and high affinity for receptors on reticulocyte membrane suggested the participation of the proteins evaluated here in some steps related to host cell invasion. Using recombinant proteins and reticulocytes in host-pathogen interaction has provided one of the best alternatives to date^21, 22, 81^.

Bearing in mind that obtaining pure reticulocytes in high amounts for binding assays is extremely difficult due to their low concentration in blood and little stability, UCB RBC or reticulocyte-depleted normocytes were here used for characterizing specific *Pv*AMA-1 reticulocyte binding properties using three different receptor-ligand interaction techniques. Rosette formation led to recombinant expression in a mammalian system containing all the machinery for the correct *P. vivax* protein folding. Flow cytometry was used for determining which specific RBC population each recombinant protein assayed here bound to and which short regions might be mediating such binding. Finally, a robust methodology was used for confirming peptide-cell interaction specificity and determining physicochemical parameters.

Binding assays using the aforementioned methodologies (including soluble recombinant proteins and proteins expressed on COS-7 cell membrane) showed that *Pv*AMA-DI-II recognized a receptor just on CD71^+^ reticulocytes (i.e. a *P. vivax* target cell) (Fig. 1 and 2). Receptor-ligand interaction was affected by treating UCB RBC with chymotrypsin and neuraminidase. The same enzymatic profile observed for *Pv*AMA-DI-II has been described for glycophorin B which is a receptor for erythrocyte binding ligand-1 (EBL-1)^12^ and the receptor for Rh1 binding; however, future experiments should explore the identity of the *Pv*AMA-DI-II receptor. “Why do only AMA-1 domains I and II bind to CD71^+^ reticulocytes if the full *Pv*AMA-1 ectodomain contains domains I-II-III?” represents an interesting question which emerged from the binding assays. Miss-folding could explain this pattern; however, these fragments were expressed in COS-7 ensuring correct protein folding (Fig. 1). mAb F8.12.19 which recognized a conformational epitope in DIII^65^ recognized both complete r*Pv*AMA-1 and r*Pv*AMA-DII-III (Fig. 2a). Some studies have shown that different AMA-1 recombinant domain combinations (i.e. DI-DII or DIII only) maintain the same structure as the complete AMA-1 protein^32^.

A previous study found that *Pf*AMA-1 domain III was the only fragment which could specifically interact with a receptor on RBC membrane, only becoming exposed when RBC were treated with trypsin, while the complete ectodomain did not interact. The authors suggested that this might have been due to *Pf*AMA-1 cleavage and that DIII acts as a receptor for invasion^38^. Even though the presence of fragments has not been determined for *Pv*AMA-1, it cannot be ruled out that the protein undergoes proteolytic processing similar to that for *Pf*AMA-1 and that the resulting fragments play a role in binding. A similar pattern has been described for *Pf*MSP-1 and its cleavage products^8, 82^.

It has been suggested that AMA-1 participates in the invasion stage which is conserved for most apicomplexa, as well as having similar tertiary structure in *Plasmodium spp*.^60^; however, different binding properties have been reported for its homologues. It has been reported through rosetting assays that different *Pf*AMA-1 domains did not bind to intact human RBC^38^; however, DIII have bound to human erythrocytes pre-treated with trypsin^38^ while *Py*AMA-1 DI-DII has only interacted with intact mouse and rat erythrocytes but not with trypsin- or chymotrypsin-treated erythrocytes^37^. Complementing other authors’ reports^32^, the results presented here suggested that AMA-1 recognizes specific receptors on different *Plasmodium* host cells in close relationship with target cell preference.

Comparative analysis of the proteome of human RBC and reticulocytes has shown that membrane or cytosol protein detected thereby was not unique to just erythrocytes or reticulocytes^83^. The difference between these two cells thus lying in the abundance of each protein, having around 500 membrane proteins, being lower in erythrocytes than in reticulocytes^83^. It seems that such differences could be critical for *P. vivax* to have tropism regarding reticulocytes compared to normocytes; however, it is still not clear whether *P. vivax* selectivity for its target cells could be just mediated during initial invasion stages where low affinity interactions occur, or whether this happens during late stages where high affinity interactions are established or simply that selectivity is maintained throughout reticulocyte invasion.

When defining domain I and II regions responsible for binding to CD71^+^ reticulocytes it was found that peptide 21270 located between residues^81^E to^100^ G in domain I significantly inhibited the soluble recombinant protein (r*Pv*AMA-DI-II) binding and rosette formation, strongly suggesting that this peptide has *Pv*AMA-1 binding properties. Furthermore, to reinforce this inhibition experiment, it was found that peptide 21270 is a HABP which binds specifically and with high affinity to reticulocytes, having an enzyme binding profile similar to that reported for the soluble recombinant protein (r*Pv*AMA-DI-II). Interestingly, this peptide’s amino acid sequence is conserved among strains having various geographical origins. Several studies have shown that AMA-1’s polymorphic nature prevents it being considered a vaccine candidate^61^; in fact, the results of different *Pf*AMA-1 formulations assayed at clinical level have shown that a protective response has been strain specific. Thus, finding conserved regions of *Pv*AMA-1 having a binding function could be used for resolving one of the parasite’s main evasion mechanisms: antigenic polymorphism^84^. No binding peptides were found in *Pv*AMA-1 domain II; it has been found in other species, such as *P. falciparum*, that AMA-1 domain II does not participate in binding to RBC^38^ but does play a role during *Pf*RON2 interaction-related invasion^16, 85^. Structural studies have suggested that DII protects a significant portion of the binding site in AMA-1 against a host’s immune response and can be readily displaced to extend the hydrophobic groove for effective binding to *Pf*RON2^16^. Such structural changes regarding AMA-1-RON2 interaction have been used for explaining invasion inhibiting antibodies (i.e. 4G2) which have recognised the base of a loop in *Pf*AMA-1 ectodomain DII^43^. MAb 4G2 binding has prevented DII loop displacement for effective *Pf*RON2 binding, thereby affecting tight junction formation during parasite invasion and thus inhibiting invasion^16^. Even though such interactions have not been studied in *P. vivax*, *Pv*AMA-1 DII participation in invasion cannot be ruled out.

A methodology based on identifying conserved sequences having high specific host cell binding capability has been used with *P. falciparum* and other microorganisms such as *Mycobacterium tuberculosis* during the last 25 years for characterizing conserved HABPs (cHABP) directly involved in target cell binding^73, 86^.

Regarding *P. falciparum* it has been described that many cHABPs form niches or clefts where they or the receptors can bind^87^. For example, *Pf*AMA-1 cHABPs 4313 (^134^DAEVAGTQYRLPSGKCPVFG^153^) and 4325 (^374^MIKSAFLPTGAFKADRYKSH^393^) formed a channel where a yet-to-be characterized receptor bound during Mrz invasion. *P. falciparum* circumsporozoite (*Pf*CSP) cHABP 4397 formed a hydrophobic cavity where hepatocyte heparan sulphate proteoglycans (HSPG) bound, while cHABP 3279, located in the *P. falciparum* thrombospondin-related anonymous protein (*Pf*TRAP) von Willebrand domain, folded to create a cavity where the cholesterol molecule bound during Spz invasion^87^.

Previous studies have highlighted AMA-1’s role as adhesin during invasion^36^. It has been described recently that *P. falciparum* and *T. gondii* AMA-1 DI-II domains interact with a RON2-derived protein which might be mediating TJ formation^16, 88^. Structural and inhibition studies regarding this interaction have supported the argument concerning TJ/complex formation being crucial for parasite entry^16, 40^. However, AMA-1 conditional knockout studies have shown that modified parasites can form the characteristic TJ ring^89^, suggesting varied roles for AMA-1 during invasion. The present work thus found that *Pv*AMA-1 domain I and II were interacting with reticulocytes, i.e. acting as an adhesin.

Two *P. vivax* cHABPs were identified here (both located in *Pv*AMA-1 domain I) having structural differences regarding their localization; cHABP 21270 formed part of the trough formed between PAN domains and (as in *Pf*AMA-1) could be the critical pocket where the receptor binds. Out of the 8 HABPs identified previously in the whole *Pf*AMA-1 ectodomain^36^, cHABP 4313 was a homolog of cHABP 21270, having 95% similarity. Interestingly, cHABP 4313 inhibited Mrz invasion of erythrocytes by 75%^36^ suggesting that this region´s critical role during this stage of the cycle. However, this region does not participate as adhesin during Spz invasion of hepatocytes; rather, the parasite uses different *Pf*AMA-1 regions to interact^90^. *P. vivax* cHABP 21268 was located on the same face as HABP 21270 but opposite the trough; even though this cHABP did not inhibit r*Pv*AMA-DI-II binding by flow cytometry in 1:20 molar ratio, it displayed a low inhibition of rosette formation (by 25%) (Supplementary Fig. S4).

Bearing cHABP 21268 location in mind and its low inhibitory capacity, it can be suggested that, even though it might specifically interact with a receptor on reticulocyte membrane, it is not the determinant region in protein binding. When *Pv*AMA-1 polymorphisms were located on the 3D structure, it was found that most were located on the opposite face of the identified cHABPs, suggesting that one face of *Pv*AMA-1 is more exposed to the immune system than others^60^. It has been reported that different microorganisms hide relevant regions from the immune system while exposing immunodominant (but non-functional) regions under immune pressure, to protect antigen function during invasion^91^.

To date, *Pv*MSP-1^19^, *Pv*RBP-1a^92^ and *Pv*DBP^72^ HABPs have been identified which bind specifically to reticulocytes; this, together with characterizing *Pv*AMA-1 regions, has increased current knowledge about *P. vivax* Mrz specific binding properties used for invading reticulocytes. Future work should address evaluating naturally-acquired antibodies capability for blocking *Pv*AMA-1 binding to reticulocytes and correlate the results with some degree of protection. Future work should also be aimed at identifying critical binding residues which could be modified (mHABP) to increase cHABP immunogenicity and protection-inducing capability, following the methodology proposed for developing a vaccine against *P. falciparum* ^93^.

## Methods

### Constructing recombinant plasmids

Three *Pv*AMA-1 fragments corresponding to the ectodomain (residues 43-487), domain I and II (residues 43-386) and domain II and III (249-487) were amplified from *Plasmodium vivax* VCG-I strain genomic DNA for *Pv*AMA-1 expression as a recombinant protein and expression on COS-7 cells (American Type Culture Collection CRL-1651). Gene specific primers were designed using the *Pv*AMA-1 genomic sequence (PVX_092275) available in PlasmoDB^93^ as template. Each fragment was amplified using a KAPA HiFi HotStart ReadyMix PCR kit at 25 μL final reaction volume, containing 12.5 μL 2x KAPA HiFi Ready Mix, 1.5 μL of each primer (Supplementary Table S1) at 5 μM concentration and 7.5 μL of nuclease-free water. The amplification conditions for the three products consisted of one 5-min cycle at 95 °C followed by 35 cycles lasting 20 sec at 98 °C, 45 sec at 56 °C and 2 min at 72 °C with a final extension cycle lasting 5 min at 72 °C. Purified products were digested with DraI or PvuII and ApaI restriction enzymes and cloned in pRE4 vector^64^ in frame with the HSVgD signal sequence and transmembrane segment^17^ to express these fragments on COS-7 cells membrane or digested with BamHI and SalI and cloned in pQE30 vector in frame with histidine tag at the N-terminal to be expressed in *E. coli*. Each construct was transformed in JM109 cells and selected on Luria Bertani (LB) plates containing ampicillin and confirmed by sequencing. The resulting plasmids were labelled pRE4-*Pv*AMA-1, pRE4-*Pv*AMA-DI-II, pRE4-*Pv*AMA-DII-III, pQE30-*Pv*AMA-1, pQE30-*Pv*AMA-DI-II and pQE30-*Pv*AMA-DII-III.

### COS-7 cell culture and transfection

COS-7 cells were cultured in Dulbecco-Modified Eagle Medium (DMEM) with 10% FCS in a humidified 5% CO_2_ incubator at 37 °C. For all transfection experiments, 3 × 10^4^ cells were seeded in 3.5-cm diameter wells (40–60% confluent) and then transfected with 300 ng of each recombinant plasmid using FuGENE HD transfection reagent (Promega), following the manufacturer’s protocol, in the presence of OptiMEM medium for 48 h in a humidified 5% CO_2_ incubator at 37 °C. The cells were grown on coverslips for use in immunofluorescence to verify the correct protein expression on COS-7 cell surface.

### Immunofluorescence assay

Transfected cells grown on coverslips were washed in phosphate buffered saline (PBS), fixed with 2% formaldehyde (in PBS) for 15 min at room temperature (RT) and blocked with 10% FCS in PBS for 1 hr to verify correct protein expression on COS-7 cell surface. The cells were stained overnight with anti-DL6 antibodies (Santa Cruz) in 1:1,000 dilution at 4 °C which recognized a proline-rich region adjacent to the mature HSV gD protein transmembrane segment or F8.12.19 monoclonal antibody which recognized a conformational epitope in *Pv*AMA-1 domain III^65^. Fluorescein-conjugated goat anti-mouse antibodies were used as secondary antibodies. Cell nuclei were stained with 4′,6-diamidino-2-phenylindole (DAPI) and fluorescence was visualized by fluorescence microscope (Olympus BX51) using an Olympus DP2 camera and Fiji software. Transfection efficiency (%) was calculated as total amount of fluorescent COS-7 cells × 100/total amount of COS-7 cells counted in 30 fields, as described previously^67^.

### COS-7 cells used for erythrocyte binding assays

Human RBC from UCB containing about 5–7% reticulocytes were washed three times in PBS (pH 7.4) and then treated with neuraminidase, chymotrypsin or trypsin, as described earlier^94^. Briefly, an erythrocyte suspension (60% haematocrit) was incubated at 37 °C for 60 min with either neuraminidase (150 μU/mL; ICN 900-67-6), trypsin (1 mg/mL; Sigma T-1005) or chymotrypsin (1 mg/mL; Sigma C-4129). Erythrocytes were then washed twice by spinning with PBS at 2,500 × *g* for 5 min. The UCB blood used in this study came from Bogotá’s District Blood Bank (Colombia).

COS-7 cells were tested 48 h after transfection with pRE4-*Pv*AMA-1, pRE4-*Pv*AMA-DI-II or pRE4-*Pv*AMA-DII-III plasmids regarding their binding to normal, neuraminidase-treated, chymotrypsin-treated and trypsin-treated human RBC from UCB (10%) or reticulocyte-depleted erythrocytes (10% haematocrit) from peripheral blood without any treatment for 2 hr at 37 °C. Regarding competition assays, UCB (10%) was pre-incubated with 10 µL peptide 21270 (1 mg/mL) for 1 hr at 37 °C followed by two washes with incomplete DMEM and then added to transfected COS-7 cells. After incubation, the cells were washed twice with PBS. The COS-7 cells with adherent erythrocytes or normocytes (i.e. rosettes) were scored in 20 randomly-chosen fields at 200× magnification. Rosettes were considered positive when adherent erythrocytes covered more than 50% of COS-7 cell surface. COS-7 cells transfected with plasmid pHVDR22 (which expressed DBP RII) were used as positive control^17^ while non-transfected cells were used as negative control. At least three independent experiments were performed with replicates.

### Soluble recombinant expression and purification

*E. coli* (JM109) recombinant proteins transformed with pQE30-*Pv*AMA-1, pQE30-*Pv*AMA-DI-II or pQE30-*Pv*AMA-DII-III were grown in 25 mL LB broth supplemented with 0.1 mg/mL ampicillin and 0.1% D-glucose for 12 hr at 37 °C with constant shaking. This starting culture was used to inoculate 475 mL LB broth containing 0.1 mg/mL ampicillin and 0.1% D-glucose maintained at 37 °C with constant shaking until reaching 0.6–0.8 optical density (OD) at 620 nm. Recombinant protein expression was induced with isopropyl β-D1-thioglactopyranoside (IPTG) at 1 mM final concentration. The cultures were grown for another 16 hr at 30 °C and *E. coli* cells were harvested by centrifugation. The cell pellet was suspended in lysis buffer (50 mM Tris pH 8.0, 300 mM NaCl, 25 mM imidazole) supplemented with 15 mM 2-β-mercaptoethanol, 0.1 mM ethylene glycol-bis (β-aminoethyl ether)-N,N,N’,N’-tetraacetic acid (EGTA), 0.1 mg/mL lysozyme, 0.5% Tween 20 and a protease inhibitor cocktail. Bacterial cells were lysed by sonication (Torebeo Ultrasonic Processor 36800). The lysate was spun at 15,000 × g for 30 min at 4 °C and the supernatant was incubated with nickel-nitrylotriacetic acid (Ni^2+^-NTA) agarose resin for purifying recombinant proteins by affinity chromatography. Bound protein was eluted with increasing imidazole gradients (50–500 mM). The eluates were analyzed on SDS-PAGE and by Western Blot and the fractions containing the recombinant protein having a clear single band were pooled. Protein concentration was determined using the BioRad protein assay system (BCA method) and a standard bovine serum albumin (BSA) curve.

### ELISA and dot blot assays

The ELISA assay described elsewhere^40^ had some modifications. Briefly, 1 µg recombinant protein (r*Pv*AMA-1 or r*Pv*AMA-DII-III) was pre-absorbed onto 96-well plates in coating buffer (15 mM sodium carbonate and 35 mM sodium bicarbonate) and then blocked with 5% BSA in PBS and 1% Tween 20 at 37 °C. The plates were incubated for 1 hr with F8.12.19 monoclonal antibody in 1:16,000 dilution at 37 °C, followed by three washes with 1% PBS-Tween. Peroxidase-conjugated anti mouse IgG was used as secondary antibody, diluted 1:5,000 for 1 hr. Antigen-antibody reaction was detected using a TMB Microwell peroxidase substrate system kit (KPL Laboratories). The reaction was stopped after 10 min incubation with 1 M phosphoric acid. Absorbance was measured at 405 nm using a microtiter reader.

The dot blot assay involved seeding 10 µL r*Pv*AMA-1 or r*Pv*AMA-DII-III on nitrocellulose membrane and then incubating for 15 min in a damp chamber at 37 °C. The membrane was blocked with 5% milk in 0.5% PBS-Tween for 30 min; the membrane was then incubated with F8.12.19 monoclonal antibody (1:16,000) for 1 hr. Alkaline phosphatase conjugated anti-mouse antibody was used as secondary antibody. A BCIP/NBT (Promega) kit was used for revealing the reaction, according to the manufacturer’s instructions.

### Flow cytometry assays

The flow cytometry-based RBC-binding assay was performed as previously described, with the following modifications^21^. Twenty-five micrograms of each recombinant protein were incubated with 500 µL UCB suspension overnight at 4 °C with constant shaking. Competition assays involved adding each *Pv*AMA-1 DI- and DII-derived synthetic peptide (>99% purity) in a 1:20 (protein:peptide) molar ratio. The erythrocytes were washed with 1% BSA/PBS and then incubated with anti-human CD45 allophycocyanin (APC) (Becton Dickinson) and anti-human CD71-APC-H7 monoclonal antibodies (Becton Dickinson) for 20 min at RT following incubation with mouse anti-histidine phycoerythrin conjugated monoclonal antibody (Miltenyi Biotec). RBC were washed and suspended in 2 mL PBS. One million events were read on a FACSCanto II (BD Bioscience) and the results were analyzed using FlowJo software (TreeStar). DBPRII recombinant soluble protein was used as positive binding control and DBPRIII-IV as negative control. At least three independent experiments were performed with different UCB samples.

### *Pv*AMA-1 peptide synthesis and radio-labeling

*Pv*AMA-1 domain I and II (PVX_092275) were synthesized in sequential peptides (20-mer) using the t-Boc amino-acid strategy and p methylbenzhydrylamine resin (0.5 mequiv/g, Bachem), following solid-phase multiple peptide synthesis methodology^95, 96^. Peptides were cleaved by the low–high hydrogen fluoride technique and analyzed by RP-HPLC and MALDI-TOF mass spectrometry (Bruker Daltonics). Conserved peptides were radiolabeled using 3 µL -Na^125^I (100 mCi/mL; ARC) and 15 µL chloramine T (2.75 mg/mL). The reaction was stopped after 15 min by adding 15 µL sodium metabisulphite (2.3 mg/mL), as described previously^97^. Radiolabeled peptides were separated by size-exclusion chromatography on a Sephadex G-25 column (Pharmacia). Each eluted fraction was then analyzed by gamma counter (Packard Cobra II).

### Radiolabeled peptide binding assays

2 × 10^7^ RBC from UCB were incubated at RT for 90 min with different concentrations of each radiolabeled peptide (0–800 nM) in the absence (total binding) or presence (non-specific binding) of the same unlabeled peptide dissolved in 4-(2-hydroxyethyl)-1-piperazineethanesulphonic acid buffered saline (HBS; 200 μL final volume). Following incubation, cells were washed twice with HBS and the amount of cell-bound radiolabeled peptide was quantified on an automatic gamma counter.

Peptides’ specific binding activity was evaluated in a binding assay with enzyme-treated RBC from UCB. The RBC were treated as described in the COS-7 cell RBC binding assay. 2 × 10^7^ untreated RBC and trypsin-, chymotrypsin- or neuraminidase-treated RBC were incubated at RT for 90 min with 400 nM of each radiolabeled peptide in the absence or presence of the same unlabelled peptide, as described previously^97^.

### Determining dissociation constants

Modified binding assays were used for determining HABP 21270 dissociation constants (*K*_d_), and Hill coefficients (*n*H). Briefly, 1.5 × 10^7^ RBC were incubated with increasing concentrations of radiolabeled peptide (0–4,000 nM) in the absence or presence of unlabelled peptide (20 μM), using a 255 μL final volume. Cell-bound radioactivity was quantified as in binding assays^97^. GraphPad Prism software v5 was used for calculating *K*_d_ and *n*H, following an interaction model, one by one.

### Statistical analysis

SPSS v20.0 was used for statistical analysis of RBC-binding assays. Comparative statistics were reported from non-parametric univariate analysis. Differences between means were compared by Kruskal-Wallis tests when comparing multiple groups or Mann-Whitney U-test for comparing two groups.

### Ethics approval and consent to participate

All parents from the donors of umbilical cord samples here used signed an informed consent form after receiving detailed information regarding the study’s goals. The umbilical cord samples were collected by Bogotá’s District Blood Centre (Colombia), following the protocols established by this institution, in accordance with Colombian laws and regulations. All procedures were approved by FIDIC’s ethics committee.

## Electronic supplementary material

Supplementary information
