# Supplementary material for: Plasmodium vivax ligand-receptor interaction: PvAMA-1 domain I contains the minimal regions for specific interaction with CD71+ reticulocytes
Source: Sci Rep. 2017 Aug 30;7:9616. doi: 10.1038/s41598-017-10025-6 (PMC5577344; doi:10.1038/s41598-017-10025-6)
Supplement: Supplementary file 1 — Supplementary information [file 41598_2017_10025_MOESM1_ESM.pdf]

Supplementary Information

Plasmodium vivax ligand-receptor interaction: *Pv*AMA-1 domain I contains the minimal regions for specific interaction with $CD71^+$ reticulocytes

Gabriela Arévalo-Pinzón [1, 2, +], Maritza Bermúdez [1, 3, +], Diana Hernández [1], Hernando Curtidor [1, 4], Manuel Alfonso Patarroyo [1, 4 *]

[1] Fundación Instituto de Inmunología de Colombia (FIDIC), Carrera 50 # 26-20, Bogotá, Colombia.

[2] PhD Program in Biomedical and Biological Sciences, Universidad del Rosario, Carrera 24 # 63C-69, Bogotá, Colombia.

[3] MSc Program in Biological Sciences, Pontificia Universidad Javeriana, Carrera 7 # 40-62, Bogotá, Colombia.

[4] School of Medicine and Health Sciences, Universidad del Rosario, Carrera 24 # 63C-69, Bogotá, Colombia.

+Equal contributors

Supplementary Table S1. A list of the primers used for amplifying *Pv*AMA-1 fragments. The restriction sites for each one are highlighted in bold.

Recombinant fragments	Primers	Used	Sequence
*Pv*AMA1	Ectodo 1	Heterologous Expression (*E. coli*)	5’-CG**G GAT CC**C CTA CCG TTG AGA GAA GCA-3’
	Ectodo 2		5’-ACG C**GT CGA C**TA GTA GCA TCT GCT TGT TCG-3’
*Pv*DI-II	Ectodo 1		5’-CG**G GAT CC**C CTA CCG TTG AGA GAA GCA-3’
	DI-DIIR		5’-ACG C**GT CGAC** TTA CTC CAG GTC TAC TTC TTG-3’
*Pv*DII-DIII	DII-DIIIF		5’-CG**G GAT CC**C GTA AAA ATT TAG GAA ACG CC-3’
	Ectodo 2		5’-ACG C**GT CGA C**TA GTA GCA TCT GCT TGT TCG-3’
*Pv*AMA1	AMA-1Cos-7F	Expression on COS7 cells	5’-GAA **TTT AAA** CCTA CCG TTG AGA GAA GC-3’
	AMA-1Cos-7R		5’-GAT **GGG CCC** TAG TAG CAT CTG CTT GTT CG-3’
*Pv*DI-II	AMA-1Cos-7F		5’-GAA **TTT AAA** CCTA CCG TTG AGA GAA GC-3’
	AMA-1Cos-7EctoI-IIR		5’-GCA **GGG CCC** CTC CAG GTC TAC TTC TTG-3’
*Pv*DII-DIII	AMA-1Cos-7EctoII-IIIF		5’-AGC **CAG CTG** GTG CGT AAT GAT TGG GAT AAA-3’
	AMA-1cos-7R		5’-GAT **GGG CCC** TAG TAG CAT CTG CTT GTT CG-3’

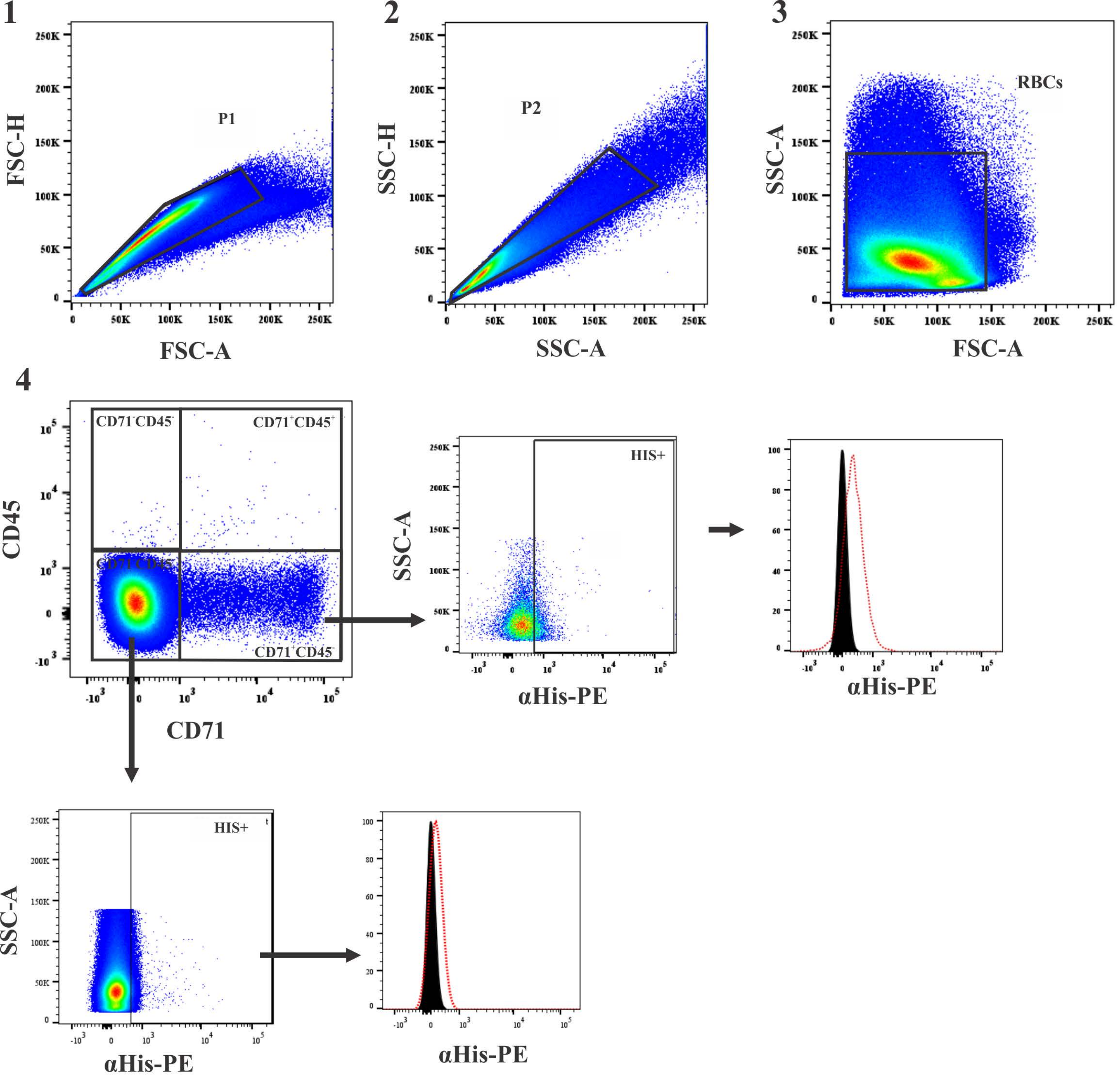

Figure S1. Flow chart used for selecting the study population by flow cytometry. Gating the general population regarding acquired events (P1, P2) for selecting RBC. Data was analyzed regarding CD45 labeling compared to CD71. Recombinant binding was compared to that of PE conjugated anti-histidine antibody binding to $CD71^{+}CD45^{-}$ (reticulocytes) or $CD71^{-}CD45^{-}$ cells (normocytes and older reticulocytes).

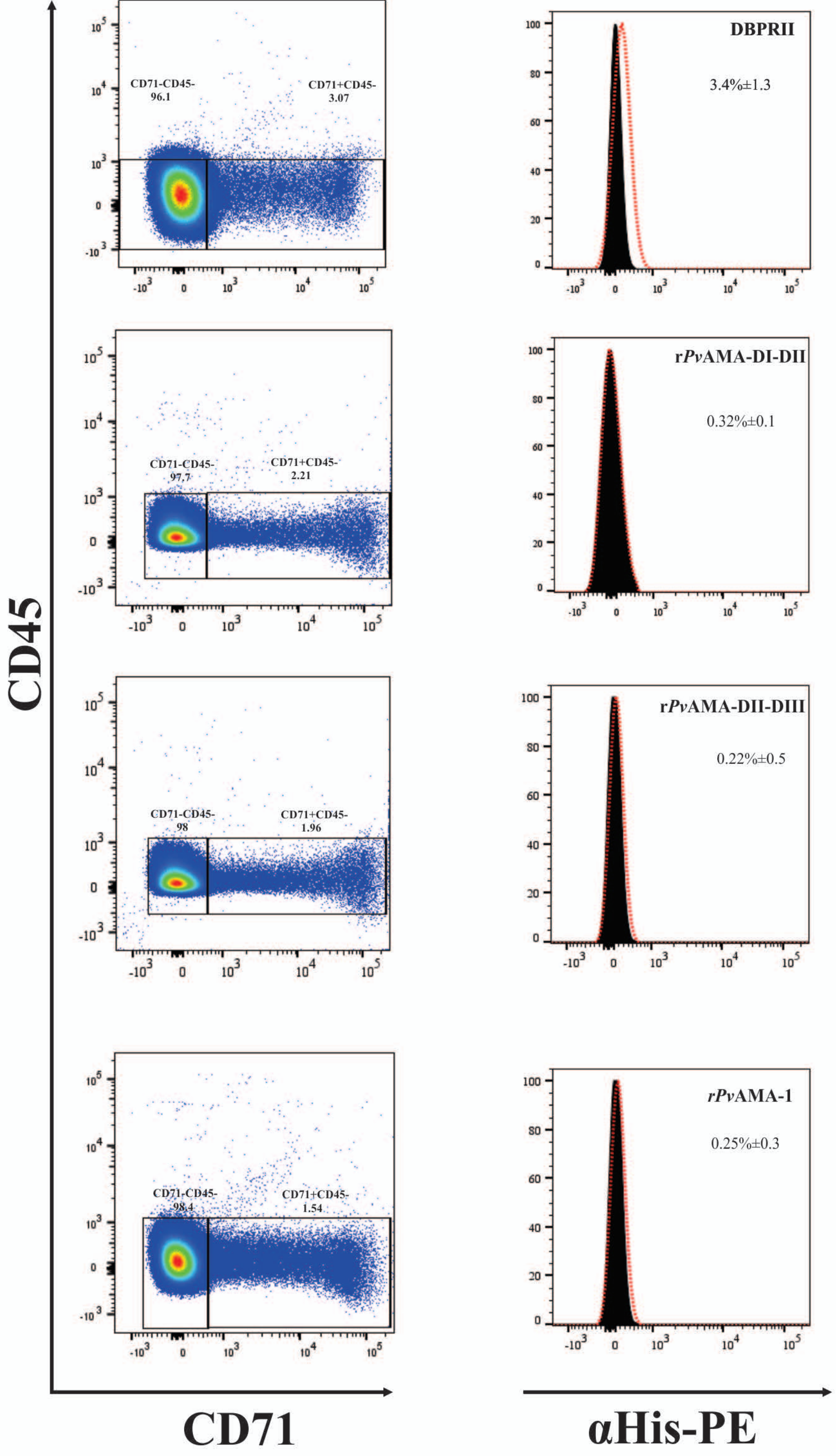

Figure S2. Flow cytometry CD71⁻CD45⁻ cell binding experiments. Binding to normocytes was determined by gating the CD71-CD45- population. CD71+CD45- cell binding percentages for each protein are shown on the X axis.

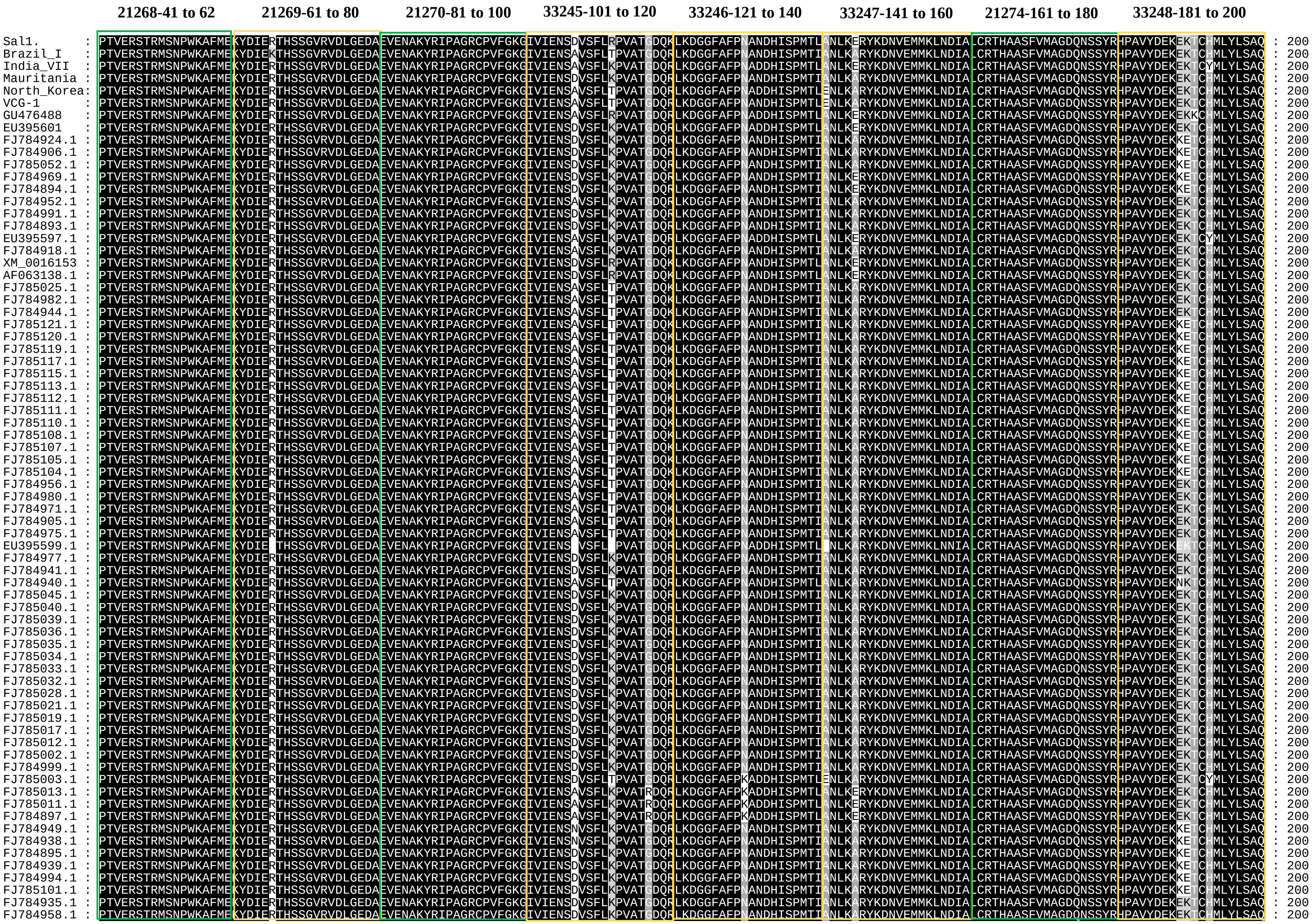
21268-41 to 62
21269-61 to 80
21270-81 to 100
33245-101 to 120
33246-121 to 140
33247-141 to 160
21274-161 to 180
33248-181 to 200

```
FJ784947.1 : PTVERSTRMSNPWKAFMEKYDIERTHSSGVRVDLGEDAEVENAKYRIPAGRCPVFGKGIVIENSAVSFLKPVATGDQRLKDGGFAFPKADDHISPMTLANLKERYKDNVEMMKLNDIALCRTHAASFVMAGDQNSSYRHPAVYDEKEKTCHMLYLSAQ : 200
FJ784948.1 : PTVERSTRMSNPWKAFMEKYDIERTHSSGVRVDLGEDAEVENAKYRIPAGRCPVFGKGIVIENSAVSFLTPVATGDQRLKDGGFAFPKADDHISPMTIANLKARYKDNVEMMKLNDIALCRTHAASFVMAGDQNSSYRHPAVYDEKEKTCHMLYLSAQ : 200
FJ785023.1 : PTVERSTRMSNPWKAFMEKYDIERTHSSGVRVDLGEDAEVENAKYRIPAGRCPVFGKGIVIENSAVSFLTPVATGDQRLKDGGFAFPKADDHISPMTLANLKERYKDNVEMMKLNDIALCRTHAASFVMAGDQNSSYRHPAVYDEKEKTCHMLYLSAQ : 200
FJ785010.1 : PTVERSTRMSNPWKAFMEKYDIERTHSSGVRVDLGEDAEVENAKYRIPAGRCPVFGKGIVIENSAVSFLTPVATGDQRLKDGGFAFPKADDHISPMTLANLKERYKDNVEMMKLNDIALCRTHAASFVMAGDQNSSYRHPAVYDEKEKTCHMLYLSAQ : 200
FJ785006.1 : PTVERSTRMSNPWKAFMEKYDIERTHSSGVRVDLGEDAEVENAKYRIPAGRCPVFGKGIVIENSAVSFLTPVATGDQRLKDGGFAFPKADDHISPMTLANLKERYKDNVEMMKLNDIALCRTHAASFVMAGDQNSSYRHPAVYDEKEKTCHMLYLSAQ : 200
FJ784996.1 : PTVERSTRMSNPWKAFMEKYDIERTHSSGVRVDLGEDAEVENAKYRIPAGRCPVFGKGIVIENSAVSFLTPVATGDQRLKDGGFAFPKADDHISPMTLANLKERYKDNVEMMKLNDIALCRTHAASFVMAGDQNSSYRHPAVYDEKEKTCHMLYLSAQ : 200
FJ784993.1 : PTVERSTRMSNPWKAFMEKYDIERTHSSGVRVDLGEDAEVENAKYRIPAGRCPVFGKGIVIENSAVSFLTPVATGDQRLKDGGFAFPKADDHISPMTLANLKERYKDNVEMMKLNDIALCRTHAASFVMAGDQNSSYRHPAVYDEKEKTCHMLYLSAQ : 200
FJ785007.1 : PTVERSTRMSNPWKAFMEKYDIERTHSSGVRVDLGEDAEVENAKYRIPAGRCPVFGKGIVIENSAVSFLKPVATGDQRLKDGGFAFPNANDHISPMTIANLKARYKDNVEMMKLNDIALCRTHAASFVMAGDQNSSYRHPAVYDEKEKTCHMLYLSAQ : 200
FJ784914.1 : PTVERSTRMSNPWKAFMEKYDIERTHSSGVRVDLGEDAEVENAKYRIPAGRCPVFGKGIVIENSDVSFLKPVATGDQRLKDGGFAFPNANDHISPMTIANLKARYKDNVEMMKLNDIALCRTHAASFVMAGDQNSSYRHPAVYDEKEKTCHMLYLSAQ : 200
FJ784896.1 : PTVERSTRMSNPWKAFMEKYDIERTHSSGVRVDLGEDAEVENAKYRIPAGRCPVFGKGIVIENSDVSFLKPVATGDQRLKDGGFAFPNANDHISPMTIANLKARYKDNVEMMKLNDIALCRTHAASFVMAGDQNSSYRHPAVYDEKEKTCHMLYLSAQ : 200
FJ784934.1 : PTVERSTRMSNPWKAFMEKYDIERTHSSGVRVDLGEDAEVENAKYRIPAGRCPVFGKGIVIENSAVSFLKPVATGDQRLKDGGFAFPNANDHISPMTIANLKARYKDNVEMMKLNDIALCRTHAASFVMAGDQNSSYRHPAVYDEKEKTCHMLYLSAQ : 200
FJ784926.1 : PTVERSTRMSNPWKAFMEKYDIERTHSSGVRVDLGEDAEVENAKYRIPAGRCPVFGKGIVIENSAVSFLKPVATGDQRLKDGGFAFPNANDHISPMTIANLKARYKDNVEMMKLNDIALCRTHAASFVMAGDQNSSYRHPAVYDEKEKTCHMLYLSAQ : 200
FJ785118.1 : PTVERSTRMSNPWKAFMEKYDIERTHSSGVRVDLGEDAEVENAKYRIPAGRCPVFGKGIVIENSAVSFLKPVATGDQRLKDGGFAFPNANDHISPMTIANLKARYKDNVEMMKLNDIALCRTHAASFVMAGDQNSSYRHPAVYDEKEKTCHMLYLSAQ : 200
FJ785116.1 : PTVERSTRMSNPWKAFMEKYDIERTHSSGVRVDLGEDAEVENAKYRIPAGRCPVFGKGIVIENSAVSFLKPVATGDQRLKDGGFAFPNANDHISPMTIANLKARYKDNVEMMKLNDIALCRTHAASFVMAGDQNSSYRHPAVYDEKEKTCHMLYLSAQ : 200
FJ785114.1 : PTVERSTRMSNPWKAFMEKYDIERTHSSGVRVDLGEDAEVENAKYRIPAGRCPVFGKGIVIENSAVSFLKPVATGDQRLKDGGFAFPNANDHISPMTIANLKARYKDNVEMMKLNDIALCRTHAASFVMAGDQNSSYRHPAVYDEKEKTCHMLYLSAQ : 200
FJ785109.1 : PTVERSTRMSNPWKAFMEKYDIERTHSSGVRVDLGEDAEVENAKYRIPAGRCPVFGKGIVIENSAVSFLKPVATGDQRLKDGGFAFPNANDHISPMTIANLKARYKDNVEMMKLNDIALCRTHAASFVMAGDQNSSYRHPAVYDEKEKTCHMLYLSAQ : 200
FJ785106.1 : PTVERSTRMSNPWKAFMEKYDIERTHSSGVRVDLGEDAEVENAKYRIPAGRCPVFGKGIVIENSAVSFLKPVATGDQRLKDGGFAFPNANDHISPMTIANLKARYKDNVEMMKLNDIALCRTHAASFVMAGDQNSSYRHPAVYDEKEKTCHMLYLSAQ : 200
FJ784990.1 : PTVERSTRMSNPWKAFMEKYDIERTHSSGVRVDLGEDAEVENAKYRIPAGRCPVFGKGIVIENSNVSFLTPVATGDQRLKDGGFAFPNANDHISPMTIANLKARYKDNVEMMKLNDIALCRTHAASFVMAGDQNSSYRHPAVYDEKEKTCHMLYLSAQ : 200
FJ784987.1 : PTVERSTRMSNPWKAFMEKYDIERTHSSGVRVDLGEDAEVENAKYRIPAGRCPVFGKGIVIENSNVSFLTPVATGDQRLKDGGFAFPNANDHISPMTIANLKARYKDNVEMMKLNDIALCRTHAASFVMAGDQNSSYRHPAVYDEKEKTCHMLYLSAQ : 200
FJ784986.1 : PTVERSTRMSNPWKAFMEKYDIERTHSSGVRVDLGEDAEVENAKYRIPAGRCPVFGKGIVIENSNVSFLTPVATGDQRLKDGGFAFPNANDHISPMTIANLKARYKDNVEMMKLNDIALCRTHAASFVMAGDQNSSYRHPAVYDEKEKTCHMLYLSAQ : 200
FJ784984.1 : PTVERSTRMSNPWKAFMEKYDIERTHSSGVRVDLGEDAEVENAKYRIPAGRCPVFGKGIVIENSAVSFLKPVATGDQRLKDGGFAFPNANDHISPMTIANLKARYKDNVEMMKLNDIALCRTHAASFVMAGDQNSSYRHPAVYDEKEKTCHMLYLSAQ : 200
FJ784970.1 : PTVERSTRMSNPWKAFMEKYDIERTHSSGVRVDLGEDAEVENAKYRIPAGRCPVFGKGIVIENSAVSFLKPVATGDQRLKDGGFAFPNANDHISPMTIANLKARYKDNVEMMKLNDIALCRTHAASFVMAGDQNSSYRHPAVYDEKEKTCHMLYLSAQ : 200
FJ784925.1 : PTVERSTRMSNPWKAFMEKYDIERTHSSGVRVDLGEDAEVENAKYRIPAGRCPVFGKGIVIENSAVSFLTPVATGDQRLKDGGFAFPNANDHISPMTLENLKARYKDNVEMMKLNDIALCRTHAASFVMAGDQNSSYRHPAVYDEKEKTCHMLYLSAQ : 200
FJ784928.1 : PTVERSTRMSNPWKAFMEKYDIERTHSSGVRVDLGEDAEVENAKYRIPAGRCPVFGKGIVIENSAVSFLKPVATGDQRLKDGGFAFPNANDHISPMTLENLKARYKDNVEMMKLNDIALCRTHAASFVMAGDQNSSYRHPAVYDEKEKTCHMLYLSAQ : 200
FJ785014.1 : PTVERSTRMSNPWKAFMEKYDIERTHSSGVRVDLGEDAEVENAKYRIPAGRCPVFGKGIVIENSAVSFLKPVATGDQRLKDGGFAFPNANDHISPMTLENLKARYKDNVEMMKLNDIALCRTHAASFVMAGDQNSSYRHPAVYDEKEKTCHMLYLSAQ : 200
EU395595.1 : PTVERSTRMSNPWKAFMEKYDIERTHSSGVRVDLGEDAEVENAKYRIPAGRCPVFGKGIVIENSAVSFLKPVATGDQRLKDGGFAFPNANDHISPMTLENLKARYKDNVEMMKLNDIALCRTHAASFVMAGDQNSSYRHPAVYDEKEKTCHMLYLSAQ : 200
EU395600.1 : PTVERSTRMSNPWKAFMEKYDIERTHSSGVRVDLGEDAEVENAKYRIPAGRCPVFGKGIVIENSAVSFLKPVATGDQRLKDGGFAFPNANDHISPMTLENLKARYKDNVEMMKLNDIALCRTHAASFVMAGDQNSSYRHPAVYDEKEKTCHMLYLSAQ : 200
FJ784917.1 : PTVERSTRMSNPWKAFMEKYDIERTHSSGVRVDLGEDAEVENAKYRIPAGRCPVFGKGIVIENSDVSFLKPVATGDQRLKDGGFAFPNANDHISPMTLENLKARYKDNVEMMKLNDIALCRTHAASFVMAGDQNSSYRHPAVYDEKEKTCHMLYLSAQ : 200
FJ784953.1 : PTVERSTRMSNPWKAFMEKYDIERTHSSGVRVDLGEDAEVENAKYRIPAGRCPVFGKGIVIENSDVSFLKPVATGDQRLKDGGFAFPNANDHISPMTIANLKARYKDNVEMMKLNDIALCRTHAASFVMAGDQNSSYRHPAVYDEKEKTCHMLYLSAQ : 200
FJ784979.1 : PTVERSTRMSNPWKAFMEKYDIERTHSSGVRVDLGEDAEVENAKYRIPAGRCPVFGKGIVIENSDVSFLKPVATGDQRLKDGGFAFPNANDHISPMTIANLKARYKDNVEMMKLNDIALCRTHAASFVMAGDQNSSYRHPAVYDEKEKTCHMLYLSAQ : 200
FJ784967.1 : PTVERSTRMSNPWKAFMEKYDIERTHSSGVRVDLGEDAEVENAKYRIPAGRCPVFGKGIVIENSDVSFLKPVATGDQRLKDGGFAFPNANDHISPMTIANLKARYKDNVEMMKLNDIALCRTHAASFVMAGDQNSSYRHPAVYDEKEKTCHMLYLSAQ : 200
FJ784964.1 : PTVERSTRMSNPWKAFMEKYDIERTHSSGVRVDLGEDAEVENAKYRIPAGRCPVFGKGIVIENSDVSFLKPVATGDQRLKDGGFAFPNANDHISPMTIANLKARYKDNVEMMKLNDIALCRTHAASFVMAGDQNSSYRHPAVYDEKEKTCHMLYLSAQ : 200
FJ784936.1 : PTVERSTRMSNPWKAFMEKYDIERTHSSGVRVDLGEDAEVENAKYRIPAGRCPVFGKGIVIENSDVSFLKPVATGDQRLKDGGFAFPNANDHISPMTIANLKARYKDNVEMMKLNDIALCRTHAASFVMAGDQNSSYRHPAVYDEKEKTCHMLYLSAQ : 200
FJ784954.1 : PTVERSTRMSNPWKAFMEKYDIERTHSSGVRVDLGEDAEVENAKYRIPAGRCPVFGKGIVIENSNVSFLKPVATGDQRLKDGGFAFPNANDHISPMTIANLKARYKDNVEMMKLNDIALCRTHAASFVMAGDQNSSYRHPAVYDEKEKTCHMLYLSAQ : 200
```

```
             33249-201 to 220    21282-221 to 240    33250-241 to 260    21284-261 to 280    21285-281 to 300    21286-301 to 320    21287-321 to 340    21288-341 to 360
Sal1.       : ENMGPRYCSPDAQNRDAVFCFKPDKNESFENLVYLSKNVRNDWDKKCPRKNLGNAKFGLWVDGNCEEIPYVKEVEAEDLRECNRIVFGASASDQPTQYEEEMTDYQKIQQGFRQNNREMIKSAFLPVGAFNSDNFKSKGRGFNWANFDSVKKKCYIFN : 358
Brazil_I    : ENMGPRYCSPDAQNRDAVFCFKPDKNESFENLVYLSKNVRNDWDKKCPRKNLGNAKFGLWVDGNCEEIPYVKEVEAKDLRECNRIVFGASASDQPTQYEEEMTDYQKIQQGFRQNNREMIKSAFLPVGAFNSDNFKSKGRGFNWANFDSVKKKCYIFN : 358
India_VII   : ENMGPRYCSSDAQNRDAVFCFKPDKNESFENLVYLSKNVRNDWDKKCPRKNLGNAKFGLWVDGNCEEIPYVKEVEAKDLRECNRIVFGASASDQPTQYEEEMTDYQKIQQGFRQNNREMIKSAFLPVGAFNSDNFKSKGRGFNWANFDSVKKKCYIFN : 358
Mauritania  : ENMGPRYCSPDAQNRDAVFCFKPDKNESFENLVYLSKNVRNDWDKKCPRKNLGNAKFGLWVDGNCEEIPYVKEVEAKDLRECNRIVFGASASDQPTQYEEEMTDYQKIQQGFRQNNREMIKSAFLPVGAFNSDNFKSKGRGFNWANFDSVKKKCYIFN : 358
North_Korea : ENMGPRYCSPDAQNRDAVFCFKPDKNESFENLVYLSKNVRNDWDKKCPRKNLGNAKFGLWVDGNCEEIPYVKEVEAKDLRECNRIVFGASASDQPTQYEEEMTDYQKIQQGFRQNNREMIKSAFLPVGAFNSDNFKSKGRGFNWANFDSVKKKCYIFN : 358
VCG-1       : ENMGPRYCSPDAQNRDAVFCFKPDKNESFENLVYLSKNVRNDWDKKCPRKNLGNAKFGLWVDGNCEEIPYVKEVEAKDLRECNRIVFGASASDQPTQYEEEMTDYQKIQQGFRQNNREMIKSAFLPVGAFNSDNFKSKGRGFNWANFDSVKNKCYIFN : 358
GU476488    : ENMGPRYCSPDAQNRDAVFCFKPDKNVDFENLVYLSKNVRNDWDKKCPRKNLGNAKFGLWVDGNCEEIPYVKEVEAKDLRECNRIVFGASASDQPTQYEEEMTDYQKIQQGFRQNNREMIKSAFLPVGAFNSDNFKSKGRGFNWANFDSVKKKCYIFN : 358
EU395601    : ENMGPRYCSPDAQNRDAVFCFKPGKNVDFENLVYLSKNVRNDWDKKCPRKNLGNAKFGLWVDGNCEEIPYVKEVEAKDLRECNRIVFGASASDQPTQYEEEMTDYQKIQQGFRQNNREMIKSAFLPVGAFNSDNFKSKGRGFNWANFDSVKKKCYIFN : 358
FJ784924.1  : ENMGPRYCSSDAQNRDAVFCFKPDKNVDFENLVYLSKNVRNDWDKKCPRKNLGNAKFGLWVDGNCEEIPYVKEVEAKDLRECNRIVFGASASDQPTQYEEEMTDYQKIQQGFRQNNREMIKSAFLPVGAFNSDNFKSKGRGFNWANFDSVKNKCYIFN : 358
FJ784906.1  : ENMGPRYCSSDAQNRDAVFCFKPDKNVDFENLVYLSKNVRNDWDKKCPRKNLGNAKFGLWVDGNCEEIPYVKEVEAKDLRECNRIVFGASASDQPTQYEEEMTDYQKIQQGFRQNNREMIKSAFLPVGAFNSDNFKSKGRGFNWANFDSVKKKCYIFN : 358
FJ785052.1  : ENMGPRYCSSDAQNRDAVFCFKPDKNVDFENLVYLSKNVRNDWDKKCPRKNLGNAKFGLWVDGNCEEIPYVKEVEAEDLRECNRIVFGASASDQPTQYEEEMTDYQKIQQGFRQNNREMIKSAFLPVGAFNSDNFKSKGRGFNWANFDSVKKKCYIFN : 358
FJ784969.1  : ENMGPRYCSSDAQNRDAVFCFKPDKNVDFENLVYLSKNVRNDWDKKCPRKNLGNAKFGLWVDGNCEEIPYVKEVEAKDLRECNRIVFGASASDQPTQYEEEMTDYQKIQQGFRQNNREMIKSAFLPVGAFNSDNFKSKGRGFNWANFDSVKKKCYIFN : 358
FJ784894.1  : ENMGPRYCSSDAQNRDAVFCFKPDKNVDFENLVYLSKNVRNDWDKKCPRKNLGNAKFGLWVDGNCEEIPYVKEVEAKDLRECNRIVFGASASDQPTQYEEEMTDYQKIQQGFRQNNREMIKSAFLPVGAFNSDNFKSKGRGFNWANFDSVKKKCYIFN : 358
FJ784952.1  : ENMGPRYCSSDAQNRDAVFCFKPDKNVDFENLVYLSKNVRNDWDKKCPRKNLGNAKFGLWVDGNCEEIPYVKEVEAEDLRECNRIVFGASASDQPTQYEEEMTDYQKIQQGFRQNNREMIKSAFLPVGAFNSDNFKSKGRGFNWANFDSVKKKCYIFN : 358
FJ784991.1  : ENMGPRYCSPDAQNRDAVFCFKPDKNVDFENLVYLSKNVRNDWDKKCPRKNLGNAKFGLWVDGNCEEIPYVKEVEAEDLRECNRIVFGASASDQPTQYEEEMTDYQKIQQGFRQNNREMIKSAFLPVGAFNSDNFKSKGRGFNWANFDSVKKKCYIFN : 358
FJ784893.1  : ENMGPRYCSSDAQNRDAVFCFKPDKNVDFENLVYLSKNVRNDWDKKCPRKNLGNAKFGLWVDGNCEEIPYVKEVEAKDLRECNRIVFEASASDQPTQYEEEMTDYQKIQQGFRQNNREMIKSAFLPVGAFNSDNFKSKGRGFNWANFDSVKKKCYIFN : 358
EU395597.1  : ENMGPRYCSSDAQNRDAVFCFKPDKNESFENLVYLSKNVRNDWDKKCPRKNLGNAKFGLWVDGNCEEIPYVKEVEAKDLRECNRIVFGASASDQPTQYEEEMTDYQKIQQGFRQNNREMIKSAFLPVGAFNSDNFKSKGRGFNWANFDSVKKKCYIFN : 358
FJ784918.1  : ENMGPRYCSSDAQNRDAVFCFKPDKNESFENLVYLSKNVRNDWDKKCPRKNLGNAKFGLWVDGNCEEIPYVKEVEAEDLRECNRIVFGASASDQPTQYEEEMTDYQKIQQGFRQNNREMIKSAFLPVGAFNSDNFKSKGRGFNWANFDSVKNKCYIFN : 358
XM_0016153  : ENMGPRYCSPDAQNRDAVFCFKPDKNESFENLVYLSKNVRNDWDKKCPRKNLGNAKFGLWVDGNCEEIPYVKEVEAEDLRECNRIVFGASASDQPTQYEEEMTDYQKIQQGFRQNNREMIKSAFLPVGAFNSDNFKSKGRGFNWANFDSVKKKCYIFN : 358
AF063138.1  : ENMGPRYCSPDAQNRDAVFCFKPDKNESFENLVYLSKNVRNDWDKKCPRKNLGNAKFGLWVDGNCEEIPYVKEVEAEDLRECNRIVFGASASDQPTQYEEEMTDYQKIQQGFRQNNREMIKSAFLPVGAFNSDNFKSKGRGFNWANFDSVKKKCYIFN : 358
FJ785025.1  : ENMGPRYCSSDAQNRDAVFCFKPDKNESFENLVYLSKNVRNDWDKKCPRKNLGNAKFGLWVDGNCEEIPYVKEVEAEDLRECNRIVFGASASDQPTQYEEEMTDYQKIQQGFRQNNREMIKSAFLPVGAFNSDNFKSKGRGFNWANFDSVKKKCYIFN : 358
FJ784982.1  : ENMGPRYCSSDAQNRDAVFCFKPDKNESFENLVYLSKNVRNDWDKKCPRKNLGNAKFGLWVDGNCEEIPYVKEVEAEDLRECNRIVFGASASDQPTQYEEEMTDYQKIQQGFRQNNREMIKSAFLPVGAFNSDNFKSKGRGFNWANFDSVKKKCYIFN : 358
FJ784944.1  : ENMGPRYCSSDAQNRDAVFCFKPDKNESFENLVYLSKNVRNDWDKKCPRKNLGNAKFGLWVDGNCEEIPYVKEVEAEDLRECNRIVFGASASDQPTQYEEEMTDYQKIQQGFRQNNREMIKSAFLPVGAFNSDNFKSKGRGFNWANFDSVKKKCYIFN : 358
FJ785121.1  : ENMGPRYCSPDAQNRDAVFCFKPDKNESFENLVYLSKNVRNDWDKKCPRKNLGNAKFGLWVDGNCEEIPYVKEVEAKDLRECNRIVFEASASDQPTQYEEEMTDYQKIQQGFRQNNREMIKSAFLPVGAFNSDNFKSKGRGFNWANFDSVKKKCYIFN : 358
FJ785120.1  : ENMGPRYCSPDAQNRDAVFCFKPDKNESFENLVYLSKNVRNDWDKKCPRKNLGNAKFGLWVDGNCEEIPYVKEVEAKDLRECNRIVFEASASDQPTQYEEEMTDYQKIQQGFRQNNREMIKSAFLPVGAFNSDNFKSKGRGFNWANFDSVKKKCYIFN : 358
FJ785119.1  : ENMGPRYCSPDAQNRDAVFCFKPDKNESFENLVYLSKNVRNDWDKKCPRKNLGNAKFGLWVDGNCEEIPYVKEVEAKDLRECNRIVFEASASDQPTQYEEEMTDYQKIQQGFRQNNREMIKSAFLPVGAFNSDNFKSKGRGFNWANFDSVKKKCYIFN : 358
FJ785117.1  : ENMGPRYCSPDAQNRDAVFCFKPDKNESFENLVYLSKNVRNDWDKKCPRKNLGNAKFGLWVDGNCEEIPYVKEVEAKDLRECNRIVFEASASDQPTQYEEEMTDYQKIQQGFRQNNREMIKSAFLPVGAFNSDNFKSKGRGFNWANFDSVKKKCYIFN : 358
FJ785115.1  : ENMGPRYCSPDAQNRDAVFCFKPDKNESFENLVYLSKNVRNDWDKKCPRKNLGNAKFGLWVDGNCEEIPYVKEVEAKDLRECNRIVFEASASDQPTQYEEEMTDYQKIQQGFRQNNREMIKSAFLPVGAFNSDNFKSKGRGFNWANFDSVKKKCYIFN : 358
FJ785113.1  : ENMGPRYCSPDAQNRDAVFCFKPDKNESFENLVYLSKNVRNDWDKKCPRKNLGNAKFGLWVDGNCEEIPYVKEVEAKDLRECNRIVFEASASDQPTQYEEEMTDYQKIQQGFRQNNREMIKSAFLPVGAFNSDNFKSKGRGFNWANFDSVKKKCYIFN : 358
FJ785112.1  : ENMGPRYCSPDAQNRDAVFCFKPDKNESFENLVYLSKNVRNDWDKKCPRKNLGNAKFGLWVDGNCEEIPYVKEVEAKDLRECNRIVFEASASDQPTQYEEEMTDYQKIQQGFRQNNREMIKSAFLPVGAFNSDNFKSKGRGFNWANFDSVKKKCYIFN : 358
FJ785111.1  : ENMGPRYCSPDAQNRDAVFCFKPDKNESFENLVYLSKNVRNDWDKKCPRKNLGNAKFGLWVDGNCEEIPYVKEVEAKDLRECNRIVFEASASDQPTQYEEEMTDYQKIQQGFRQNNREMIKSAFLPVGAFNSDNFKSKGRGFNWANFDSVKKKCYIFN : 358
FJ785110.1  : ENMGPRYCSPDAQNRDAVFCFKPDKNESFENLVYLSKNVRNDWDKKCPRKNLGNAKFGLWVDGNCEEIPYVKEVEAKDLRECNRIVFEASASDQPTQYEEEMTDYQKIQQGFRQNNREMIKSAFLPVGAFNSDNFKSKGRGFNWANFDSVKKKCYIFN : 358
FJ785108.1  : ENMGPRYCSPDAQNRDAVFCFKPDKNESFENLVYLSKNVRNDWDKKCPRKNLGNAKFGLWVDGNCEEIPYVKEVEAKDLRECNRIVFEASASDQPTQYEEEMTDYQKIQQGFRQNNREMIKSAFLPVGAFNSDNFKSKGRGFNWANFDSVKKKCYIFN : 358
FJ785107.1  : ENMGPRYCSPDAQNRDAVFCFKPDKNESFENLVYLSKNVRNDWDKKCPRKNLGNAKFGLWVDGNCEEIPYVKEVEAKDLRECNRIVFEASASDQPTQYEEEMTDYQKIQQGFRQNNREMIKSAFLPVGAFNSDNFKSKGRGFNWANFDSVKKKCYIFN : 358
FJ785105.1  : ENMGPRYCSPDAQNRDAVFCFKPDKNESFENLVYLSKNVRNDWDKKCPRKNLGNAKFGLWVDGNCEEIPYVKEVEAKDLRECNRIVFEASASDQPTQYEEEMTDYQKIQQGFRQNNREMIKSAFLPVGAFNSDNFKSKGRGFNWANFDSVKKKCYIFN : 358
FJ785104.1  : ENMGPRYCSPDAQNRDAVFCFKPDKNESFENLVYLSKNVRNDWDKKCPRKNLGNAKFGLWVDGNCEEIPYVKEVEAKDLRECNRIVFEASASDQPTQYEEEMTDYQKIQQGFRQNNREMIKSAFLPVGAFNSDNFKSKGRGFNWANFDSVKKKCYIFN : 358
FJ784956.1  : ENMGPRYCSPDAQNRDAVFCFKPDKNESFENLVYLSKNVRNDWDKKCPRKNLGNAKFGLWVDGNCEEIPYVKEVEAKDLRECNRIVFEASASDQPTQYEEEMTDYQKIQQGFRQNNREMIKSAFLPVGAFNSDNFKSKGRGFNWANFDSVKKKCYIFN : 358
FJ784980.1  : ENMGPRYCSPDAQNRDAVFCFKPDKNESFENLVYLSKNVRNDWDKKCPRKNLGNAKFGLWVDGNCEEIPYVKEVEAKDLRECNRIVFEASASDQPTQYEEEMTDYQKIQQGFRQNNREMIKSAFLPVGAFNSDNFKSKGRGFNWANFDSVKEKCYIFN : 358
FJ784971.1  : ENMGPRYCSPDAQNRDAVFCFKPDKNESFENLVYLSKNVRNDWDKKCPRKNLGNAKFGLWVDGNCEEIPYVKEVEAKDLRECNRIVFEASASDQPTQYEEEMTDYQKIQQGFRQNNREMIKSAFLPVGAFNSDNFKSKGRGFNWANFDSVKEKCYIFN : 358
FJ784905.1  : ENMGPRYCSPDAQNRDAVFCFKPDKNESFENLVYLSKNVRNDWDKKCPRKNLGNAKFGLWVDGNCEEIPYVKEVEAKDLRECNRIVFEASASDQPTQYEEEMTDYQKIQQGFRQNNREMIKSAFLPVGAFNSDNFKSKGRGFNWANFDSVKEKCYIFN : 358
FJ784975.1  : ENMGPRYCSPDAQNRDAVFCFKPDKNESFENLVYLSKNVRNDWDKKCPRKNLGNAKFGLWVDGNCEEIPYVKEVEAKDLRECNRIVFEASASDQPTQYEEEMTDYQKIQQGFRQNNREMIKSAFLPVGAFNSDNFKSKGRGFNWANFDSVKEKCYIFN : 358
EU395599.1  : ENMGPRYCSPDAQNRDAVFCFKPDKNESFENLVYLSKNVRNDWDKKCPRKNLGNAKFGLWVDGNCEEIPYVKEVEAKDLRECNRIVFGASASDQPTQYEEEMTDYQKIQQGFRQNNREMIKSAFLPVGAFNSDNFKSKGRGFNWANFDSVKKKCYIFN : 358
FJ784977.1  : ENMGPRYCSPDAQNRDAVFCFKPDKNESFENLVYLSKNVRNDWDKKCPRKNLGNAKFGLWVDGNCEEIPYVKEVEAKDLRECNRIVFEASASDQPTQYEEEMTDYQKIQQGFRQNNREMIKSAFLPVGAFNSDNFKSKGRGFNWANFDSVKKKCYIFN : 358
FJ784941.1  : ENMGPRYCSPDAQNRDAVFCFKPDKNESFENLVYLSKNVRNDWDKKCPRKNLGNAKFGLWVDGNCEEIPYVKEVEAKDLRECNRIVFEASASDQPTQYEEEMTDYQKIQQGFRQNNREMIKSAFLPVGAFNSDNFKSKGRGFNWANFDSVKKKCYIFN : 358
FJ784940.1  : ENMGPRYCSPDAQNRDAVFCFKPDKNESFENLVYLSKNVRNDWDKKCPRKNLGNAKFGLWVDGNCEEIPYVKEVEAKDLRECNRIVFEASASDQPTQYEEEMTDYQKIQQGFRQNNREMIKSAFLPVGAFNSDNFKSKGRGFNWANFDSVKKKCYIFN : 358
FJ785045.1  : ENMGPRYCSSDAQNRDAVFCFKPDKNKSFENLVYLSKNVRNDWDKKCPRKNLENAKFGLWVDGNCEEIPYVKEVEAKDLRECNRIVFEASASDQPTQYEEEMTDYQKIQQGFRQNNREMIKSAFLPVGAFNSDNFKSKGRGFNWANFDSVKKKCYIFN : 358
FJ785040.1  : ENMGPRYCSSDAQNRDAVFCFKPDKNKSFENLVYLSKNVRNDWDKKCPRKNLENAKFGLWVDGNCEEIPYVKEVEAKDLRECNRIVFEASASDQPTQYEEEMTDYQKIQQGFRQNNREMIKSAFLPVGAFNSDNFKSKGRGFNWANFDSVKKKCYIFN : 358
FJ785039.1  : ENMGPRYCSSDAQNRDAVFCFKPDKNKSFENLVYLSKNVRNDWDKKCPRKNLENAKFGLWVDGNCEEIPYVKEVEAKDLRECNRIVFEASASDQPTQYEEEMTDYQKIQQGFRQNNREMIKSAFLPVGAFNSDNFKSKGRGFNWANFDSVKKKCYIFN : 358
FJ785036.1  : ENMGPRYCSSDAQNRDAVFCFKPDKNKSFENLVYLSKNVRNDWDKKCPRKNLENAKFGLWVDGNCEEIPYVKEVEAKDLRECNRIVFEASASDQPTQYEEEMTDYQKIQQGFRQNNREMIKSAFLPVGAFNSDNFKSKGRGFNWANFDSVKKKCYIFN : 358
FJ785035.1  : ENMGPRYCSSDAQNRDAVFCFKPDKNKSFENLVYLSKNVRNDWDKKCPRKNLENAKFGLWVDGNCEEIPYVKEVEAKDLRECNRIVFEASASDQPTQYEEEMTDYQKIQQGFRQNNREMIKSAFLPVGAFNSDNFKSKGRGFNWANFDSVKKKCYIFN : 358
FJ785034.1  : ENMGPRYCSSDAQNRDAVFCFKPDKNKSFENLVYLSKNVRNDWDKKCPRKNLENAKFGLWVDGNCEEIPYVKEVEAKDLRECNRIVFEASASDQPTQYEEEMTDYQKIQQGFRQNNREMIKSAFLPVGAFNSDNFKSKGRGFNWANFDSVKKKCYIFN : 358
FJ785033.1  : ENMGPRYCSSDAQNRDAVFCFKPDKNKSFENLVYLSKNVRNDWDKKCPRKNLENAKFGLWVDGNCEEIPYVKEVEAKDLRECNRIVFEASASDQPTQYEEEMTDYQKIQQGFRQNNREMIKSAFLPVGAFNSDNFKSKGRGFNWANFDSVKKKCYIFN : 358
FJ785032.1  : ENMGPRYCSSDAQNRDAVFCFKPDKNKSFENLVYLSKNVRNDWDKKCPRKNLENAKFGLWVDGNCEEIPYVKEVEAKDLRECNRIVFEASASDQPTQYEEEMTDYQKIQQGFRQNNREMIKSAFLPVGAFNSDNFKSKGRGFNWANFDSVKKKCYIFN : 358
FJ785028.1  : ENMGPRYCSSDAQNRDAVFCFKPDKNKSFENLVYLSKNVRNDWDKKCPRKNLENAKFGLWVDGNCEEIPYVKEVEAKDLRECNRIVFEASASDQPTQYEEEMTDYQKIQQGFRQNNREMIKSAFLPVGAFNSDNFKSKGRGFNWANFDSVKKKCYIFN : 358
FJ785021.1  : ENMGPRYCSSDAQNRDAVFCFKPDKNKSFENLVYLSKNVRNDWDKKCPRKNLENAKFGLWVDGNCEEIPYVKEVEAKDLRECNRIVFEASASDQPTQYEEEMTDYQKIQQGFRQNNREMIKSAFLPVGAFNSDNFKSKGRGFNWANFDSVKKKCYIFN : 358
FJ785019.1  : ENMGPRYCSSDAQNRDAVFCFKPDKNKSFENLVYLSKNVRNDWDKKCPRKNLENAKFGLWVDGNCEEIPYVKEVEAKDLRECNRIVFEASASDQPTQYEEEMTDYQKIQQGFRQNNREMIKSAFLPVGAFNSDNFKSKGRGFNWANFDSVKKKCYIFN : 358
FJ785017.1  : ENMGPRYCSSDAQNRDAVFCFKPDKNKSFENLVYLSKNVRNDWDKKCPRKNLENAKFGLWVDGNCEEIPYVKEVEAKDLRECNRIVFEASASDQPTQYEEEMTDYQKIQQGFRQNNREMIKSAFLPVGAFNSDNFKSKGRGFNWANFDSVKKKCYIFN : 358
FJ785012.1  : ENMGPRYCSSDAQNRDAVFCFKPDKNKSFENLVYLSKNVRNDWDKKCPRKNLENAKFGLWVDGNCEEIPYVKEVEAKDLRECNRIVFEASASDQPTQYEEEMTDYQKIQQGFRQNNREMIKSAFLPVGAFNSDNFKSKGRGFNWANFDSVKKKCYIFN : 358
FJ785002.1  : ENMGPRYCSSDAQNRDAVFCFKPDKNKSFENLVYLSKNVRNDWDKKCPRKNLENAKFGLWVDGNCEEIPYVKEVEAKDLRECNRIVFEASASDQPTQYEEEMTDYQKIQQGFRQNNREMIKSAFLPVGAFNSDNFKSKGRGFNWANFDSVKKKCYIFN : 358
FJ784999.1  : ENMGPRYCSSDAQNRDAVFCFKPDKNKSFENLVYLSKNVRNDWDKKCPRKNLENAKFGLWVDGNCEEIPYVKEVEAKDLRECNRIVFEASASDQPTQYEEEMTDYQKIQQGFRQNNREMIKSAFLPVGAFNSDNFKSKGRGFNWANFDSVKKKCYIFN : 358
FJ785003.1  : ENMGPRYCSPDAQNRDAVFCFKPDKNESFENLVYLSKNVRNDWDKKCPRKNLGNAKFGLWVDGNCEEIPYVKEVEAKDLRECNRIVFGASASDQPTQYEEEMTDYQKIQQGFRQNNREMIKSAFLPVGAFNSDNFKSKGRGFNWANFDSVKNKCYIFN : 358
FJ785013.1  : ENMGPRYCSPDAQNRDAVFCFKPDKNESFENLVYLSKNVRNDWDKKCPRKNLGNAKFGLWVDGNCEEIPYVKEVEAEDLRECNRIVFGASASDQPTQYEEEMTDYQKIQQGFRQNNREMIKSAFLPVGAFNSDNFKSKGRGFNWANFDSVKKKCYIFN : 358
FJ785011.1  : ENMGPRYCSPDAQNRDAVFCFKPDKNESFENLVYLSKNVRNDWDKKCPRKNLGNAKFGLWVDGNCEEIPYVKEVEAEDLRECNRIVFGASASDQPTQYEEEMTDYQKIQQGFRQNNREMIKSAFLPVGAFNSDNFKSKGRGFNWANFDSVKKKCYIFN : 358
FJ784897.1  : ENMGPRYCSPDAQNRDAVFCFKPDKNESFENLVYLSKNVRNDWDKKCPRKNLGNAKFGLWVDGNCEEIPYVKEVEAEDLRECNRIVFGASASDQPTQYEEEMTDYQKIQQGFRQNNREMIKSAFLPVGAFNSDNFKSKGRGFNWANFDSVKKKCYIFN : 358
FJ784949.1  : ENMGPRYCSSDAQNRDAVFCFKPDKNESFENLVYLSKNVRNDWDKKCPRKNLGNAKFGLWVDGNCEEIPYVKEVEAKDLRECNRIVFEASASDQPTQYEEEMTDYQKIQQGFRQNNREMIKSAFLPVGAFNSDNFKSKGRGFNWANFDSVKNKCYIFN : 358
FJ784938.1  : ENMGPRYCSPDAQNRDAVFCFKPDKNESFENLVYLSKNVRNDWDKKCPRKNLGNAKFGLWVDGNCEEIPYVKEVEAKDLRECNRIVFEASASDQPTQYEEEMTDYQKIQQGFRQNNREMIKSAFLPVGAFNSDNFKSKGRGFNWANFDSVKKKCYIFN : 358
FJ784895.1  : ENMGPRYCSSDAQNRDAVFCFKPDKNESFENLVYLSKNVRNDWDKKCPRKNLGNAKFGLWVDGNCEEIPYVKEVEAKDLRECNRIVFGASASDQPTQYEEEMTDYQKIQQGFRQNNREMIKSAFLPVGAFNSDNFKSKGRGFNWANFDSVKKKCYIFN : 358
FJ784939.1  : ENMGPRYCSSDAQNRDAVFCFKPDKNESFENLVYLSKNVRNDWDKKCPRKNLGNAKFGLWVDGNCEEIPYVKEVEAKDLRECNRIVFGASASDQPTQYEEEMTDYQKIQQGFRQNNREMIKSAFLPVGAFNSDNFKSKGRGFNWANFDSVKKKCYIFN : 358
FJ784994.1  : ENMGPRYCSSDAQNRDAVFCFKPDKNESFENLVYLSKNVRNDWDKKCPRKNLGNAKFGLWVDGNCEEIPYVKEVEAEDLRECNRIVFGASASDQPTQYEEEMTDYQKIQQGFRQNNREMIKSAFLPVGAFNSDNFKSKGRGFNWANFDSVKKKCYIFN : 358
FJ785101.1  : ENMGPRYCSSDAQNRDAVFCFKPDKNESFENLVYLSKNVRNDWDKKCPRKNLGNAKFGLWVDGNCEEIPYVKEVEAKDLRECNRIVFGASASDQPTQYEEEMTDYQKIQQGFRQNNREMIKSAFLPVGAFNSDNFKSKGRGFNWANFDSVKKKCYIFN : 358
FJ784935.1  : ENMGPRYCSPDAQNRDAVFCFKPDKNESFENLVYLSKNVRNDWDKKCPRKNLGNAKFGLWVDGNCEEIPYVKEVEAEDLRECNRIVFGASASDQPTQYEEEMTDYQKIQQGFRQNNREMIKSAFLPVGAFNSDNFKSKGRGFNWANFDSVKKKCYIFN : 358
FJ784958.1  : ENMGPRYCSSDAQNRDAVFCFKPDKNESFENLVYLSKNVRNDWDKKCPRKNLGNAKFGLWVDGNCEEIPYVKEVEAEDLRECNRIVFGASASDQPTQYEEEMTDYQKIQQGFRQNNREMIKSAFLPVGAFNSDNFKSKGRGFNWANFDSVKKKCYIFN : 358
```

```
FJ784947.1 : ENMGPRYCSSDAQNRDAVFCFKPDKNESFENLVYLSKNVRNDWDKKCPRKNLGNAKFGLWVDGNCEEIPYVKEVEAKDLRECNRIVFEASASDQPTQYEEEMTDYQKIQQGFRQNNREMIKSAFLPVGAFNSDNFKSKGRGFNWANFDSVKKKCYIFN : 358
FJ784948.1 : ENMGPRYCSPDAQNRDAVFCFKPDKNESFENLVYLSKNVRNDWDKKCPRKNLGNAKFGLWVDGNCEEIPYVKEVEAKDLRECNRIVFGASASDQPTQYEEEMTDYQKIQQGFRQNNREMIKSAFLPVGAFNSDNFKSKGRGFNWANFDSVKKKCYIFN : 358
FJ785023.1 : ENMGPRYCSPDAQNRDAVFCFKPDKNESFENLVYLSKNVRNDWDKKCPRKNLGNAKFGLWVDGNCEEIPYVKEVEAKDLRECNRIVFEASASDQPTQYEEEMTDYQKIQQGFRQNNREMIKSAFLPVGAFNSDNFKSKGRGFNWANFDSVKKKCYIFN : 358
FJ785010.1 : ENMGPRYCSPDAQNRDAVFCFKPDKNESFENLVYLSKNVRNDWDKKCPRKNLGNAKFGLWVDGNCEEIPYVKEVEAKDLRECNRIVFEASASDQPTQYEEEMTDYQKIQQGFRQNNREMIKSAFLPVGAFNSDNFKSKGRGFNWANFDSVKKKCYIFN : 358
FJ785006.1 : ENMGPRYCSPDAQNRDAVFCFKPDKNESFENLVYLSKNVRNDWDKKCPRKNLGNAKFGLWVDGNCEEIPYVKEVEAKDLRECNRIVFEASASDQPTQYEEEMTDYQKIQQGFRQNNREMIKSAFLPVGAFNSDNFKSKGRGFNWANFDSVKKKCYIFN : 358
FJ784996.1 : ENMGPRYCSPDAQNRDAVFCFKPDKNESFENLVYLSKNVRNDWDKKCPRKNLGNAKFGLWVDGNCEEIPYVKEVEAKDLRECNRIVFEASASDQPTQYEEEMTDYQKIQQGFRQNNREMIKSAFLPVGAFNSDNFKSKGRGFNWANFDSVKKKCYIFN : 358
FJ784993.1 : ENMGPRYCSSDAQNRDAVFCFKPDKNESFENLVYLSKNVRNDWDKKCPRKNLGNAKFGLWVDGNCEEIPYVKEVEAKDLRECNRIVFGASASDQPTQYEEEMTDYQKIQQGFRQNNREMIKSAFLPVGAFNSDNFKSKGRGFNWANFDSVKKKCYIFN : 358
FJ785007.1 : ENMGPRYCSPDAQNRDAVFCFKPDKNESFENLVYLSKNVRNDWDKKCPRKNLGNAKFGLWVDGNCEEIPYVKEVEAKDLRECNRIVFEASASDQPTQYEEEMTDYQKIQQGFRQNNREMIKSAFLPVGAFNSDNFKSKGRGFNWANFDSVKKKCYIFN : 358
FJ784914.1 : ENMGPRYCSSDAQNRDAVFCFKPDKNKSFENLVYLSKNVRNDWDKKCPRKNLGNAKFGLWVDGNCEEIPYVKEVEAKDLRECNRIVFEASASDQPTQYEEEMTDYQKIQQGFRQNNREMIKSAFLPVGAFNSDNFKSKGRGFNWANFDSVKNKCYIFN : 358
FJ784896.1 : ENMGPRYCSSDAQNRDAVFCFKPDKNKSFENLVYLSKNVRNDWDKKCPRKNLGNAKFGLWVDGNCEEIPYVKEVEAKDLRECNRIVFEASASDQPTQYEEEMTDYQKIQQGFRQNNREMIKSAFLPVGAFNSDNFKSKGRGFNWANFDSVKKKCYIFN : 358
FJ784934.1 : ENMGPRYCSSDAQNRDALFCFKPDKNESFENLVYLSKNVRNDWDKKCPRKNLGNAKFGLWVDGNCEEIPYVKEVEAKDLRECNRIVFGASASDQPTQYEEEMTDYQKIQQGFRQNNREMIKSAFLPVGAFNSDNFKSKGRGFNWANFDSVKKKCYIFN : 358
FJ784926.1 : ENMGPRYCSSDAQNRDALFCFKPDKNESFENLVYLSKNVRNDWDKKCPRKNLGNAKFGLWVDGNCEEIPYVKEVEAKDLRECNRIVFGASASDQPTQYEEEMTDYQKIQQGFRQNNREMIKSAFLPVGAFNSDNFKSKGRGFNWANFDSVKKKCYIFN : 358
FJ785118.1 : ENMGPRYCSSDAQNRDALFCFKPDKNESFENLVYLSKNVRNDWDKKCPRKNLGNAKFGLWVDGNCEEIPYVKEVEAKDLRECNRIVFGASASDQPTQYEEEMTDYQKIQQGFRQNNREMIKSAFLPVGAFNSDNFKSKGRGFNWANFDSVKNKCYIFN : 358
FJ785116.1 : ENMGPRYCSSDAQNRDALFCFKPDKNESFENLVYLSKNVRNDWDKKCPRKNLGNAKFGLWVDGNCEEIPYVKEVEAKDLRECNRIVFGASASDQPTQYEEEMTDYQKIQQGFRQNNREMIKSAFLPVGAFNSDNFKSKGRGFNWANFDSVKNKCYIFN : 358
FJ785114.1 : ENMGPRYCSSDAQNRDALFCFKPDKNESFENLVYLSKNVRNDWDKKCPRKNLGNAKFGLWVDGNCEEIPYVKEVEAKDLRECNRIVFGASASDQPTQYEEEMTDYQKIQQGFRQNNREMIKSAFLPVGAFNSDNFKSKGRGFNWANFDSVKNKCYIFN : 358
FJ785109.1 : ENMGPRYCSSDAQNRDALFCFKPDKNESFENLVYLSKNVRNDWDKKCPRKNLGNAKFGLWVDGNCEEIPYVKEVEAKDLRECNRIVFGASASDQPTQYEEEMTDYQKIQQGFRQNNREMIKSAFLPVGAFNSDNFKSKGRGFNWANFDSVKNKCYIFN : 358
FJ785106.1 : ENMGPRYCSSDAQNRDALFCFKPDKNESFENLVYLSKNVRNDWDKKCPRKNLGNAKFGLWVDGNCEEIPYVKEVEAKDLRECNRIVFGASASDQPTQYEEEMTDYQKIQQGFRQNNREMIKSAFLPVGAFNSDNFKSKGRGFNWANFDSVKNKCYIFN : 358
FJ784990.1 : ENMGPRYCSSDAQNRDAVFCFKPDKNESFENLVYLSKNVRNDWDKKCPRKNLGNAKFGLWVDGNCEEIPYVKEVEAKDLRECNRIVFEASASDQPTQYEEEMTDYQKIQQGFRQNNREMIKSAFLPVGAFNSDNFKSKGRGFNWANFDSVKNKCYIFN : 358
FJ784987.1 : ENMGPRYCSSDAQNRDAVFCFKPDKNESFENLVYLSKNVRNDWDKKCPRKNLGNAKFGLWVDGNCEEIPYVKEVEAKDLRECNRIVFEASASDQPTQYEEEMTDYQKIQQGFRQNNREMIKSAFLPVGAFNSDNFKSKGRGFNWANFDSVKNKCYIFN : 358
FJ784986.1 : ENMGPRYCSSDAQNRDAVFCFKPDKNESFENLVYLSKNVRNDWDKKCPRKNLGNAKFGLWVDGNCEEIPYVKEVEAKDLRECNRIVFEASASDQPTQYEEEMTDYQKIQQGFRQNNREMIKSAFLPVGAFNSDNFKSKGRGFNWANFDSVKNKCYIFN : 358
FJ784984.1 : ENMGPRYCSSDAQNRDALFCFKPDKNESFENLVYLSKNVRNDWDKKCPRKNLGNAKFGLWVDGNCEEIPYVKEVEAKDLRECNRIVFEASASDQPTQYEEEMTDYQKIQQGFRQNNREMIKSAFLPVGAFNSDNFKSKGRGFNWANFDSVKNKCYIFN : 358
FJ784970.1 : ENMGPRYCSSDAQNRDALFCFKPDKNESFENLVYLSKNVRNDWDKKCPRKNLGNAKFGLWVDGNCEEIPYVKEVEAKDLRECNRIVFEASASDQPTQYEEEMTDYQKIQQGFRQNNREMIKSAFLPVGAFNSDNFKSKGRGFNWANFDSVKNKCYIFN : 358
FJ784925.1 : ENMGPRYCSPDAQNRDAVFCFKPDKNESFENLVYLSKNVRNDWDKKCPRKNLGNAKFGLWVDGNCEEIPYVKEVEAKDLRECNRIVFGASASDQPTQYEEEMTDYQKIQQGFRQNNREMIKSAFLPVGAFNSDNFKSKGRGFNWANFDSVKKKCYIFN : 358
FJ784928.1 : ENMGPRYCSPDAQNRDAVFCFKPDKNESFENLVYLSKNVRNDWDKKCPRKNLGNAKFGLWVDGNCEEIPYVKEVEAKDLRECNRIVFGASASDQPTQYEEEMTDYQKIQQGFRQNNREMIKSAFLPVGAFNSDNFKSKGRGFNWANFDSVKKKCYIFN : 358
FJ785014.1 : ENMGPRYCSPDAQNRDAVFCFKPDKNESFENLVYLSKNVRNDWDKKCPRKNLGNAKFGLWVDGNCEEIPYVKEVEAKDLRECNRIVFEASASDQPTQYEEEMTDYQKIQQGFRQNNREMIKSAFLPVGAFNSDNFKSKGRGFNWANFDSVKKKCYIFN : 358
EU395595.1 : ENMGPRYCSPDAQNRDAVFCFKPDKNESFENLVYLSKNVRNDWDKKCPRKNLGNAKFGLWVDGNCEEIPYVKEVEAKDLRECNRIVFEASASDQPTQYEEEMTDYQKIQQGFRQNNREMIKSAFLPVGAFNSDNFKSKGRGFNWANFDSVKKKCYIFN : 358
EU395600.1 : ENMGPRYCSPDAQNRDAVFCFKPDKNESFENLVYLSKNVRNDWDKKCPRKNLGNAKFGLWVDGNCEEIPYVKEVEAKDLRECNRIVFEASASDQPTQYEEEMTDYQKIQQGFRQNNREMIKSAFLPVGAFNSDNFKSKGRGFNWANFDSVKKKCYIFN : 358
FJ784917.1 : ENMGPRYCSPDAQNRDAVFCFKPDKNESFENLVYLSKNVRNDWDKKCPRKNLGNAKFGLWVDGNCEEIPYVKEVEAKDLRECNRIVFEASASDQPTQYEEEMTDYQKIQQGFRQNNREMIKSAFLPVGAFNSDNFKSKGRGFNWANFDSVKKKCYIFN : 358
FJ784953.1 : ENMGPRYCSSDAQNRDAVFCFKPDKNESFENLVYLSKNVRNDWDKKCPRKNLGNAKFGLWVDGNCEEIPYVKEVEAKDLRECNRIVFEASASDQPTQYEEEMTDYQKIQQGFRQNNREMIKSAFLPVGAFNSDNFKSKGRGFNWANFDSVKKKCYIFN : 358
FJ784979.1 : ENMGPRYCSSDAQNRDAVFCFKPDKNESFENLVYLSKNVRNDWDKKCPRKNLGNAKFGLWVDGNCEEIPYVKEVEAKDLRECNRIVFEASASDQPTQYEEEMTDYQKIQQGFRQNNREMIKSAFLPVGAFNSDNFKSKGRGFNWANFDSVKNKCYIFN : 358
FJ784967.1 : ENMGPRYCSSDAQNRDAVFCFKPDKNESFENLVYLSKNVRNDWDKKCPRKNLGNAKFGLWVDGNCEEIPYVKEVEAKDLRECNRIVFEASASDQPTQYEEEMTDYQKIQQGFRQNNREMIKSAFLPVGAFNSDNFKSKGRGFNWANFDSVKNKCYIFN : 358
FJ784964.1 : ENMGPRYCSSDAQNRDAVFCFKPDKNESFENLVYLSKNVRNDWDKKCPRKNLGNAKFGLWVDGNCEEIPYVKEVEAKDLRECNRIVFEASASDQPTQYEEEMTDYQKIQQGFRQNNREMIKSAFLPVGAFNSDNFKSKGRGFNWANFDSVKNKCYIFN : 358
FJ784936.1 : ENMGPRYCSSDAQNRDAVFCFKPDKNESFENLVYLSKNVRNDWDKKCPRKNLGNAKFGLWVDGNCEEIPYVKEVEAKDLRECNRIVFEASASDQPTQYEEEMTDYQKIQQGFRQNNREMIKSAFLPVGAFNSDNFKSKGRGFNWANFDSVKNKCYIFN : 358
FJ784954.1 : ENMGPRYCSSDAQNRDALFCFKPDKNESFENLVYLSKNVRNDWDKKCPRKNLGNAKFGLWVDGNCEEIPYVKEVEAKDLRECNRIVFEASASDQPTQYEEEMTDYQKIQQGFRQNNREMIKSAFLPVGAFNSDNFKSKGRGFNWANFDSVKKKCYIFN : 358
```

```
              21289-361 to 380     21290-381 to 400
Sal1.       : TKPTCLINDKNFIATTALSHPQEVDLEFPCSIYKDEIEREIK  : 400
Brazil_I    : TKPTCLINDKNFIATTALSHPKEVDPEFPCSIYKDEIEREIK  : 400
India_VII   : TKPTCLINDKNFIATTALSHPQEEDPEFPCSIYKDEIEREIK- : 400
Mauritania  : TKPTCLINDKNFIATTALSHPQEEDPEFPCSIYKDEIEREIK- : 400
North_Korea : TKPTCLINDKNFIATTALSHPQEVDLEFPCSIYKDEIEREIK- : 400
VCG-1       : TKPTCLINDKNFIATTALSHPQEVDLEFPCSIYKDEIEREIK- : 400
GU476488    : TKPTCLINDKNFIATTALSHPQEVDLEFPCSIYKDEIEREIK- : 400
EU395601    : TKPTCLINDKNFIATTALSHPQEVDPEFPCSIYKDEIEREIN- : 400
FJ784924.1  : TKPTCLINDKNFIATTALSHPQEVDREFPCSIYKDEIEREIK- : 400
FJ784906.1  : TKPTCLINDKNFIATTALSHPQEVDPEFPCSIYKDEIEREIK- : 400
FJ785052.1  : TKPTCLINDKNFIATTALSHPREVDLEFPCSIYKDEIEREIK- : 400
FJ784969.1  : TKPTCLINDKNFIATTALSHPREVDLEFPCSIYKDEIEREIK- : 400
FJ784894.1  : TKPTCLINDKNFIATTALSHPREVDLEFPCSIYKDEIEREIK- : 400
FJ784952.1  : TKPTCLINDKNFIATTALSHPQEVDLEFPCSIYKDEIEREIK- : 400
FJ784991.1  : TKPTCLINDKNFIATTALSHPREVDLEFPCSIYKDEIEREIK- : 400
FJ784893.1  : TKPTCLINDKNFIATTALSHPREVDLEFPCSIYKDEIEREIK- : 400
EU395597.1  : TKPTCLINDKNFIATTALSHPQEEDPEFPCSIYKDEIEREIK- : 400
FJ784918.1  : TKPTCLINDKNFIATTALSHPQEVDPKFPCSIYKDEIEREIR- : 400
XM_0016153  : TKPTCLINDKNFIATTALSHPQEVDLEFPCSIYKDEIEREIK- : 400
AF063138.1  : TKPTCLINDKNFIATTALSHPQEVDLEFPCSIYKDEIEREIK- : 400
FJ785025.1  : TKPTCLINDKNFIATTALSHPQEVDPEFPCSIYKDEIEREIK- : 400
FJ784982.1  : TKPTCLINDKNFIATTALSHPQEVDPEFPCSIYKDEIEREIK- : 400
FJ784944.1  : TKPTCLINDKNFIATTALSHPQEVDPEFPCSIYKDEIEREIK- : 400
FJ785121.1  : TKPTCLINDKNFIATTALSHPQEVDREFPCSIYKDEIEREIK- : 400
FJ785120.1  : TKPTCLINDKNFIATTALSHPQEVDREFPCSIYKDEIEREIK- : 400
FJ785119.1  : TKPTCLINDKNFIATTALSHPQEVDREFPCSIYKDEIEREIK- : 400
FJ785117.1  : TKPTCLINDKNFIATTALSHPQEVDREFPCSIYKDEIEREIK- : 400
FJ785115.1  : TKPTCLINDKNFIATTALSHPQEVDREFPCSIYKDEIEREIK- : 400
FJ785113.1  : TKPTCLINDKNFIATTALSHPQEVDREFPCSIYKDEIEREIK- : 400
FJ785112.1  : TKPTCLINDKNFIATTALSHPQEVDREFPCSIYKDEIEREIK- : 400
FJ785111.1  : TKPTCLINDKNFIATTALSHPQEVDREFPCSIYKDEIEREIK- : 400
FJ785110.1  : TKPTCLINDKNFIATTALSHPQEVDREFPCSIYKDEIEREIK- : 400
FJ785108.1  : TKPTCLINDKNFIATTALSHPQEVDREFPCSIYKDEIEREIK- : 400
FJ785107.1  : TKPTCLINDKNFIATTALSHPQEVDREFPCSIYKDEIEREIK- : 400
FJ785105.1  : TKPTCLINDKNFIATTALSHPQEVDREFPCSIYKDEIEREIK- : 400
FJ785104.1  : TKPTCLINDKNFIATTALSHPQEVDREFPCSIYKDEIEREIK- : 400
FJ784956.1  : TKPTCLINDKNFIATTALSHPQEVDLEFPCSIYKDEIEREIR- : 400
FJ784980.1  : TKPTCLINDKNFIATTALSHPQEVDREFPCSIYKDEIEREIK- : 400
FJ784971.1  : TKPTCLINDKNFIATTALSHPQEVDREFPCSIYKDEIEREIK- : 400
FJ784905.1  : TKPTCLINDKNFIATTALSHPQEVDREFPCSIYKDEIEREIK- : 400
FJ784975.1  : TKPTCLINDKNFIATTALSHPQEVDPEFPCSIYKDEIEREIK- : 400
EU395599.1  : TKPTCLINDKNFIATTALSHPQEVDLEFPCSIYKDEIEREIK- : 400
FJ784977.1  : TKPTCLINDKNFIATTALSHPQEEDPEFPCSIYKDEIEREIK- : 400
FJ784941.1  : TKPTCLINDKNFIATTALSHPQEEDPEFPCSIYKDEIEREIK- : 400
FJ784940.1  : TKPTCLINDKNFIATTALSHPQEVDLEFPCSIYKDEIEREIK- : 400
FJ785045.1  : TKPTCLINDKNFIATTALSHPQEVDPEFPCSIYKDEIEREIK- : 400
FJ785040.1  : TKPTCLINDKNFIATTALSHPQEVDPEFPCSIYKDEIEREIK- : 400
FJ785039.1  : TKPTCLINDKNFIATTALSHPQEVDPEFPCSIYKDEIEREIK- : 400
FJ785036.1  : TKPTCLINDKNFIATTALSHPQEVDPEFPCSIYKDEIEREIK- : 400
FJ785035.1  : TKPTCLINDKNFIATTALSHPQEVDPEFPCSIYKDEIEREIK- : 400
FJ785034.1  : TKPTCLINDKNFIATTALSHPQEVDPEFPCSIYKDEIEREIK- : 400
FJ785033.1  : TKPTCLINDKNFIATTALSHPQEVDPEFPCSIYKDEIEREIK- : 400
FJ785032.1  : TKPTCLINDKNFIATTALSHPQEVDPEFPCSIYKDEIEREIK- : 400
FJ785028.1  : TKPTCLINDKNFIATTALSHPQEVDPEFPCSIYKDEIEREIK- : 400
FJ785021.1  : TKPTCLINDKNFIATTALSHPQEVDPEFPCSIYKDEIEREIK- : 400
FJ785019.1  : TKPTCLINDKNFIATTALSHPQEVDPEFPCSIYKDEIEREIK- : 400
FJ785017.1  : TKPTCLINDKNFIATTALSHPQEVDPEFPCSIYKDEIEREIK- : 400
FJ785012.1  : TKPTCLINDKNFIATTALSHPQEVDPEFPCSIYKDEIEREIK- : 400
FJ785002.1  : TKPTCLINDKNFIATTALSHPQEVDPEFPCSIYKDEIEREIK- : 400
FJ784999.1  : TKPTCLINDKNFIATTALSHPQEVDPEFPCSIYKDEIEREIK- : 400
FJ785003.1  : TKPTCLINDKNFIATTALSHPQEVDLEFPCSIYKDEIEREIR- : 400
FJ785013.1  : TKPTCLINDKNFIATTALSHPQEVDLEFPCSIYKDEIEREIK- : 400
FJ785011.1  : TKPTCLINDKNFIATTALSHPQEVDLEFPCSIYKDEIEREIK- : 400
FJ784897.1  : TKPTCLINDKNFIATTALSHPQEVDLEFPCSIYKDEIEREIK- : 400
FJ784949.1  : TKPTCLINDKNFIATTALSHPQEVDPEFPCSIYKDEIEREIK- : 400
FJ784938.1  : TKPTCLINDKNFIATTALSHPQEVDRDFPCSIYKDEIEREIR- : 400
FJ784895.1  : TKPTCLINDKNFIATTALSHPQEVDRDFPCSIYKDEIEREIR- : 400
FJ784939.1  : TKPTCLINDKNFIATTALSHPQEVDPEFPCSIYKDEIEREIR- : 400
FJ784994.1  : TKPTCLINDKNFIATTALSHPQEVDLEFPCSIYKDEIEREIK- : 400
FJ785101.1  : TKPTCLINDKNFIATTALSHPREVDLEFPCSIYKDEIEREIK- : 400
FJ784935.1  : TKPTCLINDKNFIATTALSHPQEVDLEFPCSIYKDEIEREIK- : 400
```

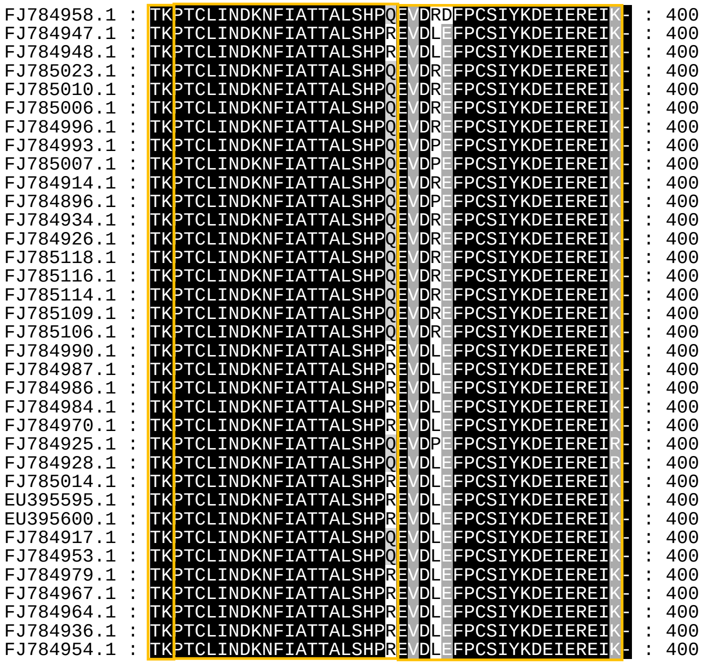

Figure S3. Aligning *Pv*AMA-1 domain I and domain II amino acids. The Sal-1 strain (PVX_092275) was taken as reference. Black highlights residues which were conserved in all the strains' and clinical isolates' sequences. The position and sequence of each synthetic peptide evaluated in inhibition and specific binding assays are shown. The yellow boxes show the variable peptides and the green ones the conserved peptides.

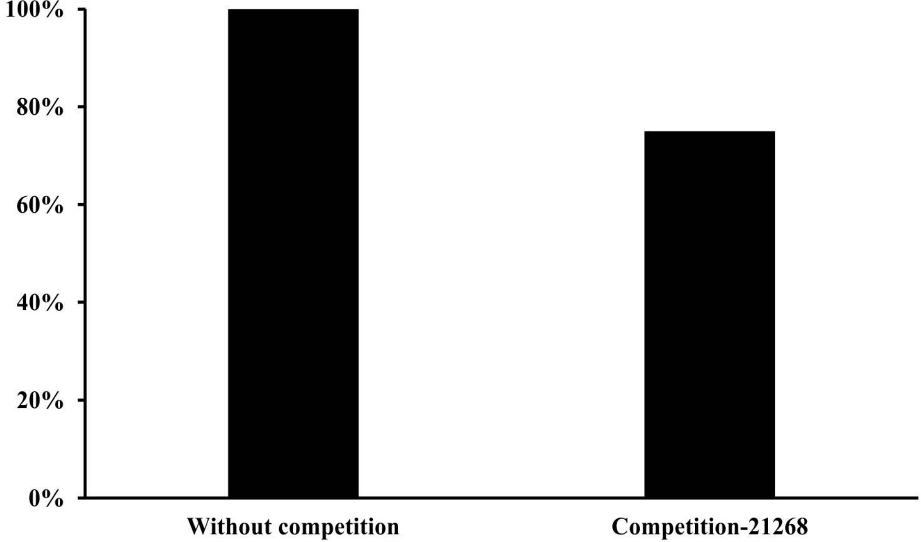

Figure S4. Rosette formation inhibition assay with peptide 21268. The values shown are the average relative percentages for *Pv*AMA-DI-II binding to UCB RBC in the absence (100%) or presence of peptide 21268. Standard deviations were below 5%.
